# Hemodynamic matrix factorization for functional magnetic resonance imaging

**DOI:** 10.1016/j.neuroimage.2021.117814

**Published:** 2021-05-01

**Authors:** Michael Hütel, Michela Antonelli, Andrew Melbourne, Sebastien Ourselin

**Affiliations:** a Department of Medical Physics and Biomedical Engineering, UCL, United Kingdom; b School of Biomedical Engineering & Imaging Sciences, KCL, United Kingdom

## Abstract

The General Linear Model (GLM) used in task-fMRI relates activated brain areas to extrinsic task conditions. The translation of resulting neural activation into a hemodynamic response is commonly approximated with a linear convolution model using a hemodynamic response function (HRF). There are two major limitations in GLM analysis. Firstly, the GLM assumes that neural activation is either on or off and matches the exact stimulus duration in the corresponding task timings. Secondly, brain networks observed in resting-state fMRI experiments present also during task experiments, but the GLM approach models these task-unrelated brain activity as noise. A novel kernel matrix factorization approach, called hemodynamic matrix factorization (HMF), is therefore proposed that addresses both limitations by assuming that task-related and task-unrelated brain activity can be modeled with the same convolution model as in GLM analysis. By contrast to the GLM, the proposed HMF is a blind source separation (BSS) technique, which decomposes fMRI data into modes. Each mode comprises of a neural activation time course and a spatial mapping. Two versions of HMF are proposed in which the neural activation time course of each mode is convolved with either the canonical HRF or predetermined subject-specific HRFs.

Firstly, HMF with the canonical HRF is applied to two open-source cohorts. These cohorts comprise of several task experiments including motor, incidental memory, spatial coherence discrimination, verbal discrimination task and a very short localization task, engaging multiple parts of the eloquent cortex. HMF modes were obtained whose neural activation time course followed original task timings and whose corresponding spatial map matched cortical areas known to be involved in the respective task processing.

Secondly, the alignment of these neural activation time courses to task timings were further improved by replacing the canonical HRF with subject-specific HRFs during HMF mode computation.

In addition to task-related modes, HMF also produced seemingly task-unrelated modes whose spatial maps matched known resting-state networks.

The validity of a fMRI task experiment relies on the assumption that the exposure to a stimulus for a given time causes an imminent increase in neural activation of equal duration. The proposed HMF is an attempt to falsify this assumption and allows to identify subject task participation that does not comply with the experiment instructions.

## 1 Introduction

Neural activation causes a complex change in neuro-physiological parameters of the cerebral blood flow (CBF) (Buxton, Uludağ, Dubowitz, Liu, 2004, Kim, Ogawa, 2012). Functional magnetic resonance imaging (fMRI) measures one of these neuro-physiological parameters, which is the blood oxygen level dependent (BOLD) response. The transition of neural activation into BOLD responses is approximated by a linear shift-invariant system (LSI) using an impulse response that is known as hemodynamic response function (HRF) (Boynton et al., 2012). In the context of fMRI, the LSI is known as hemodynamic forward model. One of the most defining assumptions of the hemodynamic forward model is that neural activity is an idealized variable, which is either on or off following the exact timing of a presented stimulus.

The most simple hemodynamic forward model convolves a fixed neural activation time course with a fixed filter (referred to as canonical HRF) known from empirical evidence (Logothetis, 2008). For a given fMRI task experiment, the hemodynamic forward model results in a BOLD time course, which is compared to observed BOLD time courses in the data to identify areas of the brain that respond to the presented stimuli of the task.

However, there is substantial intra- and inter-subject variability in the BOLD response (Aguirre, Zarahn, D’esposito, 1998, Arichi, Fagiolo, Varela, Melendez-Calderon, Allievi, Merchant, Tusor, Counsell, Burdet, Beckmann, et al., 2012, Handwerker, Gonzalez-Castillo, D’esposito, Bandettini, 2012, Handwerker, Ollinger, D’Esposito, 2004), either caused by variations in neural activation, in the HRF or in both. Therefore, estimating the variables of the hemodynamic forward model is an ill-posed problem because the BOLD response is the only observed variable, whereas neural activation and HRF are unobserved latent variables. Various approaches have been proposed that either focus on estimating neural activation or HRF by assuming either one or the other to be known.

The GLM is the most prominent model used by the approaches that estimate HRF while keeping neural activation fixed. The GLM uses the stimulus timing to construct a set of temporal regressors that are fitted to the time course of every voxel of a fMRI scan. Multiple basis functions can be used to estimate a HRF (Henson et al., 2001), usually canonical HRF and its first and second derivative are commonly used in task experiments. In general, the more basis functions are used, the more likely the GLM will overfit the data. To solve this problem, several regularization techniques have been proposed: constraints in form of temporal prior information (Ciuciu, Poline, Marrelec, Idier, Pallier, Benali, 2003, Marrelec, Benali, Ciuciu, Pélégrini-Issac, Poline, 2003), restrictions on temporal smoothness (Goutte et al., 2000) or a rank constrain on HRF estimates for similar events (Pedregosa et al., 2015). Other approaches address the regularization problem by exploiting temporal similarity among adjacent voxels. Parcellation-based HRF estimation has been introduced by Makni et al. (Makni, Beckmann, Smith, Woolrich, 2008, Makni, Ciuciu, Idier, Poline, 2005) and was subsequently extended by Vincent et al., 2010 or Chaari et al., 2013. These approaches divide the brain into temporal homogeneous regions and estimate a HRF per region.

However, the above GLM approaches rely on the assumption that evoked neural activation matches assumed neural activation in the task experiment, but it is unknown to what extent this assumption holds across the brain. More importantly, task compliance might vary greatly among subjects resulting in different neural activation patterns than intended by the experimenter. Furthermore, these approaches ignore intrinsic brain activity that operates in the background unrelated to extrinsic task processing. Such intrinsic brain activity was long considered noise before found to produce coherent spatial patterns of brain activity that are referred to as resting-state networks (Fox, Raichle, 2007, Gusnard, Raichle, 2001, Raichle, MacLeod, Snyder, Powers, Gusnard, Shulman, 2001). All the aforementioned problems occur due to the unknown nature of neural activation in task processing. In contrast to HRF estimation, blind source separation (BSS) techniques have been proposed to obtain estimates of neural activation. These techniques are part of the realm of deconvolution techniques that estimate neural activation but constrain the hemodynamic forward model to either a fix impulse function (Gaudes, Petridou, Dryden, Bai, Francis, Gowland, 2011, Glover, 1999) or a set of fix basis functions (Karahanoğlu et al., 2013a). Similarly to HRF estimation, regularization is required because the space of possible solutions is too large, multiple neural activation time courses can cause the same BOLD response time course (Rangaprakash et al., 2018). Therefore, Zarahn proposed to estimate neural activation in the time domain instead of the frequency domain using a set of limited basis functions (Zarahn, 2000). Gitelman et al. proposed to restrict these neural activation estimates with Gaussian priors (Gitelman et al., 2003).

Other deconvolution methods force neural activation estimates to be sparse (Caballero Gaudes, Petridou, Francis, Dryden, Gowland, 2013, Gaudes, Petridou, Dryden, Bai, Francis, Gowland, 2011, Hernandez-Garcia, Ulfarsson, 2011). Other approaches resemble characteristic properties of the HRF, for example, by applying sparsity regularization in the Wavelet domain with custom Wavelet basis functions (Khalidov et al., 2011).

All aforementioned approaches for neural activation estimation have been applied voxel-wise without leveraging the similarity of BOLD signal change among adjacent voxels. On the contrary, the Total Activation (TA) approach (Karahanoğlu et al., 2013a) and its extensions (Farouj, Karahanoğlu, Van De Ville, 2017, Zöller, Bolton, Karahanoğlu, Eliez, Schaer, Van De Ville, 2019) use spatial priors that enforce temporal similarity of neural activation time courses within a pre-defined region. These regions are defined by an anatomical atlas in which each voxel is assigned to only one region (or cluster).

In contrast to TA, voxels can belong to multiple clusters in mode-based approaches that decompose fMRI data into a set of spatial maps and corresponding time courses. Spatial independent component analysis (sICA) (Beckmann and Smith, 2004) is the most common mode-based approach, in which the decomposition is obtained by maximizing a proxy function for spatial independence. The main disadvantage of sICA in fMRI is that it does not incorporate spatial or temporal characteristics of fMRI data. Indeed, the order of time points of a fMRI scan can be rearranged without affecting the sICA result.

More recent mode-based approaches have therefore been proposed that model the distinctive auto-correlation in fMRI time courses and spatial correlation among adjacent voxels (Harrison, Bijsterbosch, Segerdahl, Fitzgibbon, Duff, Smith, Woolrich, 2019, Harrison, Woolrich, Robinson, Glasser, Beckmann, Jenkinson, Smith, 2015, de Pierrefeu, Löfstedt, Hadj-Selem, Dubois, Jardri, Fovet, Ciuciu, Frouin, Duchesnay, 2017, Varoquaux, Gramfort, Pedregosa, Michel, Thirion, 2011).

The brain comprises of functionally specialized regions and task activation maps revealed by fMRI experiments were therefore found to be sparse (Cole, Bassett, Power, Braver, Petersen, 2014, Friston, Price, 2001, Frost, Goebel, 2012, Petersen, Sporns, 2015). These activation maps usually comprise of multiple clusters of spatially adjacent voxels. The work by  Daubechies et al. (2009) concluded that sparsity and not independence is an ideal optimization criteria for BSS techniques in the domain of fMRI. Therefore, approaches based on sparsity and total variation regularization emerged in the literature.

The hierarchical dictionary learning approach by Varoquaux et al. (2011) incorporates a Smooth-Lasso (SL) spatial regularization term to obtain subject- and group-level spatial modes. De Pierrefeu et al. have proposed l1 sparsity and total variation (TV) constrains on spatial mode computation via PCA (de Pierrefeu et al., 2017).

Mode-based approaches have the flexibility to find different brain configurations during task processing but often do not relate BOLD signal back to original task timings.

The approach by Cherkaoui et al. (2019) derives modes that comprise of neural activation time courses and spatial maps. In contrast to mode approaches that regularize modes with respect to spatial characteristics, Cherkaoui et al. (2019) focuses on the regularization of neural activation time courses. Similarly to the TA approach,  Cherkaoui et al. (2019) uses piece-wise linear constraining on neural activation time courses but computes modes instead of the parcel-wise deconvolution performed in the TA approach.

In this work, a new mode decomposition of such kind, called Hemodynamic Matrix Factorization (HMF), is proposed that computes modes using a neural network matrix factorization framework. Each of these modes comprises of a neural activation time course and a corresponding spatial map. Two variants of HMF are proposed in which neural activation is either convolved with the canonical HRF or subject-specific pre-determined HRFs to relate observed BOLD time courses to neural activation time courses. Similarly as in de Pierrefeu et al., the spatial maps of such modes are regularized by l1 sparsity and TV (de Pierrefeu et al., 2017). The neural activation time courses are also regularized by TV which is more flexible than the discussed piece-wise constant restriction on time courses (Cherkaoui, Moreau, Halimi, Ciuciu, 2019, Karahanoğlu, Caballero-Gaudes, Lazeyras, Van De Ville, 2013a).

HMF was applied to the Midnight Scan Club (MSC) open source data^1^ that comprises of ten healthy adult subjects with five hours of task-based BOLD-fMRI experiments including motor, incidental memory, spatial coherence discrimination and verbal discrimination task, as well as to the Brainomics task data.^2^ The proposed HMF approach, blind to the original stimulus timings, recovered modes whose neural activation time course matched timings and duration of the corresponding task stimuli. Hereby, HMF revealed individual subjects whose task participation differed from anticipated experiment participation. In addition, neural activation time courses of default mode networks (DMNs) presented with an anti-correlated behavior to the task timings in MSC and Brainomics tasks.

Lastly, HMF modes were compared to modes produced by sICA, showing that BOLD time courses of HMF modes correlated stronger to the original task design than time courses produced by sICA. With respect to spatial estimates, HMF showed greater spatial reproducibility than sICA among the ten sessions of the MSC data.

## 2 Hemodynamic matrix factorization

The proposed generative model is concerned with BOLD time courses of length T, measured on V voxels: $Y\in R^{T\times V}$. The observed BOLD signal change is assumed to be driven by C latent modes that form depending on the performed task. Each mode consists of a neural activation time course $n\in R^{T\times 1}$ and a spatial map $h\in R^{1\times V}$. The corresponding BOLD time course is obtained by convolving neural activation time course n with a predetermined HRF f with length L. The neural activation time courses of all modes are aggregated in matrix $N\in R^{T\times C}$. All neural activation time courses are convolved with a predetermined HRF f by multiplying the Toeplitz matrix $F\in R^{T\times T}$ of filter f with N as depicted in Eq. (1).

(1) $$\mathrm{FN}\;=\;\left(\begin{array}{ccccc} f_{1} & 0 & \cdots & 0 & 0\\ f_{2} & f_{1} & \ldots & \vdots & \vdots \\ f_{3} & f_{2} & \ldots & 0 & 0\\ \vdots & f_{3} & \ldots & f_{1} & 0\\ f_{L-1} & \vdots & \cdots & f_{2} & f_{1}\\ f_{L} & f_{L-1} & \vdots & \vdots & f_{2}\\ 0 & f_{L} & \ldots & f_{L-2} & \vdots \\ 0 & 0 & \cdots & f_{L-1} & f_{L-2}\\ \vdots & \vdots & \vdots & f_{L} & f_{L-1}\\ 0 & 0 & 0 & \cdots & f_{L} \end{array}\right)\;\left(\begin{array}{cccc} n_{1,1} & n_{1,2} & \cdots & n_{1,C}\\ n_{2,1} & n_{2,2} & \cdots & \vdots \\ \vdots & \vdots & \cdots & \vdots \\ \vdots & \vdots & n_{T-1,C-1} & n_{T-1,C}\\ n_{T,1} & \cdots & n_{T,C-1} & n_{T,C} \end{array}\;\;\right)$$

The data Y can thus be decomposed by a matrix factorization FNH where matrix $N\in R^{T*S\times C}$ contains the individual neural activation time courses per subject $N_{s}\in R^{T\times C}$ and matrix $H\in R_{+}^{C\times V}$ contains a set of spatial maps shared among all S subjects. The loss of the proposed matrix factorization comprises of the l2 loss between original and reconstructed data, a spatial map regularization term, and a neural activation regularization term:

(2) $${\text{arg}\;\text{min}}_{N,b}\frac{1}{2}{∥M⊙\left(\mathrm{FN}\sigma ({\left(\mathrm{FN}\right)}^{⊤}Y+b)-Y\right)∥}_{2}^{2}+R_{H}+R_{N},$$

where spatial map matrix $H=\sigma ({\left(\mathrm{FN}\right)}^{⊤}Y+b)$ is computed and vector $M\in R^{1\times V}$ weighs the importance of each voxel using gray matter probabilities. The neural activation time course parameters N are initialized with a random Xavier initialization (Glorot and Bengio, 2010). The bias parameters b are initialized with zeros. The limited memory Broyden-Fletcher-Goldfarb-Shanno (L-BFGS) is used for optimizing N. The regularization terms for the spatial map matrix $R_{H}$ and neural activation matrix $R_{N}$ are introduced in the following.

### 2.1 Regularization of spatial maps

The regularization term $R_{H}=\beta_{1}R_{H_{TV}}+\beta_{2}R_{H_{S}}$ comprises of a total variation term and sparsity term. In order to compute the total variation term, the spatial map matrix H is reshaped into a 4D volume $H^{\left(4D\right)}$ to obtain the following regularization cost $R_{H_{TV}}$:

(3) $$\begin{array}{cc} R_{H_{TV}}= & \sum_{c,i,j,k}\left|H_{c,i+1,j,k}^{\left(4D\right)}-H_{c,i,j,k}^{\left(4D\right)}\right|+\\ & \left|H_{c,i,j+1,k}^{\left(4D\right)}-H_{c,i,j,k}^{\left(4D\right)}\right|+\left|H_{c,i,j,k+1}^{\left(4D\right)}-H_{c,i,j,k}^{\left(4D\right)}\right| \end{array}$$

The implementation of Eq. (3) uses an approximation that is provided in the Appendix.

The Kullback–Leibler divergence between two exponential distributions was used as regularization term for spatial sparsity. It is given by $\Delta \left(\lambda ∥{\hat{\lambda }}_{c}\right)=\log\left(\lambda \right)-\log\left({\hat{\lambda }}_{c}\right)+\frac{{\hat{\lambda }}_{c}}{\lambda }-1$ between a desired exponential distribution p with rate λ and an exponential distribution $p_{c}$ of spatial map values $H_{c}\in R^{1\times V}$ with estimated rate parameter ${\hat{\lambda }}_{c}$ resulting in the following loss term

(4) $$R_{H_{S}}=\frac{1}{C}\sum_{c=1}^{C}\Delta \left(\lambda ∥{\hat{\lambda }}_{c}\right).$$

The definition of the exponential value distribution p, the derivation of KL $\Delta (\lambda ∥{\hat{\lambda }}_{c})$ and the estimate used for ${\hat{\lambda }}_{c}$ are provided in the Appendix.

### 2.2 Regularization of neural activation

The neural activation time courses are regularized by combining l1, l2 and total variation. The l2 regularization term prevents biologically non-plausible large magnitude signal change at individual time points. The total variation and l1 regularization approximate biologically efficient neural activation organization by preferring BOLD responses produced by the most sparse and energy efficient neural activation pattern. Total variation of the neural time course matrix N is implemented by using a difference operator matrix similar to spatial regularization in Eq. (10). The complete temporal regularization term is given by

(5) $$R_{N}=\alpha_{1}∥ND_{4}{∥}_{1}+\alpha_{2}{∥N∥}_{1}+\alpha_{3}{∥N∥}_{2},$$

where $D_{4}\in R^{\left(T*S-1)\times (T*S\right)}$.

### 2.3 Canonical and subject-specific HMF

**Fig. 1:**
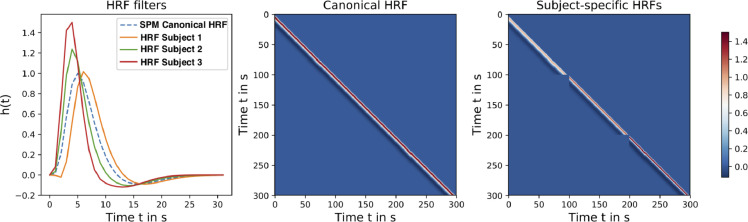
The canonical HRF and three subject-specific HRFs with distinct height and width (left). The Toeplitz matrix of the canonical HRF for three subjects concatenated in time (middle). The Toeplitz matrix of the three subject-specific HRFs in which each subject is modeled with its own HRF (right). In this example, each scan has a length of 100s.

Two variants of HMF are proposed^3^. The first is canonical HMF in which the Toeplitz matrix is built from using only the canonical HRF. The second is subject-specific HMF in which the Toeplitz matrix is built from individual HRF estimates for each subject as depicted in Fig. 1.

### 2.4 Ensemble averaging framework

**Fig. 2:**
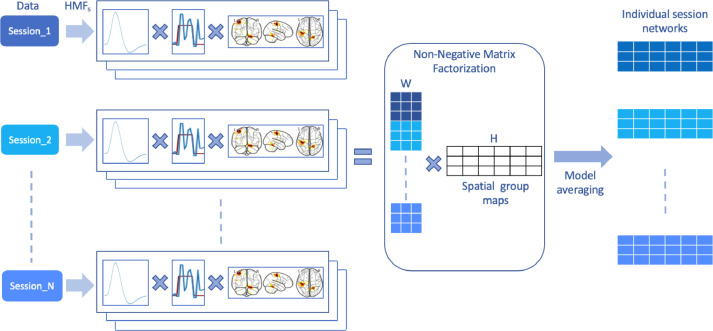
Summary of ensemble averaging of individual hemodynamic matrix factorizations to obtain individual session modes in the MSC tasks. Firstly, 10 decompositions are obtained for each task for each of the 10 sessions. All concatenated spatial maps of estimated modes are decomposed into two matrices with NMF. An association matrix and a corresponding matrix of spatial maps. The spatial maps obtained by NMF are the average of the spatial maps of all modes for a respective task. Similarly, the association matrix is used to obtain averages of neural activation time courses in the corresponding sessions.

The HMF approach is a non-convex optimization problem because of the non-linear activation function σ. Each neural activation time course is normalized by the l2 norm to address the scale ambiguity between neural activation time courses N and spatial maps H. A non-optimal solution may be obtained with only one random initialization. Therefore, several HMFs are obtained from random initializations of N. Their outputs are combined with an ensemble averaging framework as depicted in Fig. 2. An ensemble average of these HMFs across all sessions is found such that $\Omega_{opt}=\sum_{n=1}^{N}\sum_{c=1}^{C}\alpha_{n,c}\Omega_{n,c}$. A fast coordinate descent non-negative matrix factorization (CD-NMF) (Hsieh and Dhillon, 2011) is used on the concatenated spatial maps $H_{ALL}=\left\{H_{ns}\in R^{C\times V},s=1\cdots S,n=1\cdots N\right\}$ to obtain a set of group maps $H_{\mathrm{group}}$ shared across all sessions by solving for the following loss function

(6) $$\frac{1}{2}{∥AH_{group}-H_{ALL}∥}_{2}^{2}$$

A non-negative double singular value initialization (NNDSVD) (Boutsidis and Gallopoulos, 2008) is used for $A\in R^{(C*N*S)\times C}$ and $H_{group}\in R^{C\times V}$ to speed up convergence and to guarantee deterministic behavior of the ensemble averaging process. No regularization term is applied in the CD-NMF.

## 3 Materials

This section introduces the simulated data and the imaging data used to test HMF performance.

### 3.1 Simulated data

In addition to HMF evaluation, simulated data is also used to tune HMF hyper-parameters. More specifically, the simulated data is split into a training and test set, to tune the hyper-parameters of HMF and to report HMF performance, respectively.

The characteristics of HMF modes are determined by six hyper-parameters: three for spatial smoothness and sparsity $\beta =\{\beta_{1},\beta_{2},\lambda \},$ and three for total variation, l1 and l2 regularization $\alpha =\{\alpha_{1},\alpha_{2},\alpha_{3}\}$ that determine the temporal characteristics of the neural activation time courses.

In order to tune these hyper-parameters for multiple task experiments, two types of simulated fMRI data are computed. The first type resembles fast spike-like neural activation change, while the second type resembles slow block-wise neural activation. In the following, these two types are referred to as simulated event-type and block-type fMRI data, respectively.

The simulated event-type fMRI data consists of 5 brain modes that are modulated by short event-type binary neural activation, and 9 brain modes that are modulated by random neural activation drawn from a half normal distribution. Simulated block-type fMRI data consists of 5 brain modes modulated by a binary block-type neural activation, and 9 task-free modes as in the event-type data. The 14 spatial maps for these brain modes are taken from Shirer et al. (2012). Twenty training and twenty test simulated fMRI scans are generated for each type of data. Each scan is created with the generative model

**Fig. 3:**
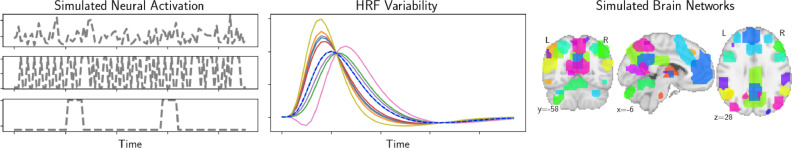
A simulated random, event and block neural activation time course depicted from top to bottom (left). Variability among the simulated HRF filters (middle). Ground truth spatial maps (right).

(7) Y=FNH+E

in which $H\in R_{+}^{C\times V}$ is the set of 14 spatial maps, $N\in R^{T\times C}$ is a matrix with either 5 event- or 5 block-type and 9 random neural activation time courses, $F\in R^{T\times T}$ is the Töplitz matrix of the individual HRF used for each subject, and $E\in R^{T\times V}$ is a Gaussian noise contribution. The original spatial maps, the subject-specific HRFs and an example of the block-type, event-type and random neural activation are depicted in Fig. 3.

#### 3.1.1 Experiments on simulated data

HMF is tested in two scenarios. In the first scenario, it is assumed that the individual HRF in each subject is unknown and therefore canonical HMF is applied. In the second scenario, the individual HRF of each subject is assumed to be known, thus, subject-specific HMF is applied. The error in neural activation estimation in the two scenarios is then compared. Fourteen HMF modes are estimated in accordance with the original number of simulated modes. For the hyper-parameters tuning, a grid-search based approach is utilized. The search cost function is the maximization of the median correlation between original and estimated neural activation time courses. This cost balances the quality of estimation in random-, event- and block-type neural activation patterns. The results are evaluated between canonical and subject-specific HMF on the test set.

### 3.2 Imaging data

The following subsections introduce the two examined fMRI data cohorts that are analyzed with HMF, the pre-processing applied to the imaging data and, finally, the conducted experiments to evaluate HMF.

#### 3.2.1 Data

Two cohorts are used to evaluate HMF: the Midnight Scan Club (MSC) (Gordon et al., 2017) and the Brainomics cohort (Orfanos, Michel, Schwartz, Pinel, Moreno, Le Bihan, Frouin, 2017, Pinel, Fauchereau, Moreno, Barbot, Lathrop, Zelenika, Le Bihan, Poline, Bourgeron, Dehaene, 2012, Pinel, Thirion, Meriaux, Jobert, Serres, Le Bihan, Poline, Dehaene, 2007). The MSC data comprises of functional and anatomical T1 images. Structural MRI was conducted across two separate days. In total, four T1-weighted images (sagittal, 224 slices, 0.8 mm isotropic resolution, TE = 3.74 ms, TR = 2400 ms, TI = 1000 ms, flip angle = 8 degrees), four T2-weighted images (sagittal, 224 slices, 0.8 mm isotropic resolution, TE = 479 ms, TR = 3200 ms) (Gordon et al., 2017). All functional imaging was performed using a gradient-echo EPI sequence (TR = 2.2 s, TE = 27 ms, flip angle = 90 degrees, voxel size = 4 mm ×4 mm ×4 mm, 36 slices) (Gordon et al., 2017).

The subjects of the MSC data were scanned in ten repetitive sessions. Each session consists of two motor task runs, two spatial discrimination task runs, two verbal discrimination task runs, and three incidental memory task runs (word discrimination, scene discrimination, face discrimination). The MSC motor task timings consist of five distinct task blocks. Each task block appears twice throughout the task. In each of these five task blocks, the subject continuously moves either left or right foot, left or right hand, or tongue. The individual task experiments of the MSC cohort are described in detail in Gordon et al., 2017.

The Brainomics data comprises of functional and anatomical T1 images. Functional images were acquired on a 3T Brucker scanner using an EPI sequence (TR = 2400 ms, TE = 30 ms, matrix size = 64×64, FOV = 24 cm ×24 cm, voxel size = 3 mm ×3 mm ×3 mm). Each volume consisted of 34 slices of 4 mm thickness. Anatomical T1 images were acquired with a spatial resolution of 1×1×1.2 mm (Pinel et al., 2007). The Brainomics cohort comprises of 94 subjects scanned with a very short and sparse paradigm to localize several areas of the eloquent cortex. The Brainomics data consists of an efficient 5 minute task protocol that maps several brain functions including auditory and visual perception, motor actions, reading, language comprehension and mental calculation (Orfanos, Michel, Schwartz, Pinel, Moreno, Le Bihan, Frouin, 2017, Pinel, Fauchereau, Moreno, Barbot, Lathrop, Zelenika, Le Bihan, Poline, Bourgeron, Dehaene, 2012, Pinel, Thirion, Meriaux, Jobert, Serres, Le Bihan, Poline, Dehaene, 2007).

#### 3.2.2 Image preparation

For the fMRI scans of each subject, volumes are realigned to the first volume to correct for head motion. The first volume is registered to its corresponding bias-corrected T1-weighted anatomical scan (affine registration). The intra-subject affine registration and non-linear inter-subject registration to the Montreal Neurological Institute (MNI) template are combined to map all fMRI volumes with one re-sampling into the MNI space. Results included in this manuscript come from pre-processing performed using FMRIPREP version 1.3.1 (Esteban et al., 2019). Time courses of voxels within a brain mask are extracted. Time courses are high-pass filtered (0.01Hz cut-off) to remove signal drifts from scanner instabilities. Time courses are centered and variance-normalized. The functional 4D volume set of each scan is reshaped into a matrix $Y\in R^{T\times V}$.

#### 3.2.3 Experiments on imaging data

The optimal hyper-parameter setting of the simulation experiments is used to compute canonical and subject-specific HMF on real imaging data. Decompositions into 40 modes are computed as motivated by empirical observations detailed in Varoquaux et al., 2010. In addition to canonical and subject-specific HMF, sICA is also applied to the imaging data. Similarly as for HMF, the gray matter segmentation in MNI space is used to weigh the importance of each voxel in sICA (Beckmann and Smith, 2004). For the Brainomics task, all subject scans are concatenated in time. One decomposition into 40 modes is computed with canonical HMF, subject-specific HMF and sICA. For each MSC task, all subject scans are concatenated in time for each session. Hence, a decomposition into 40 modes for canonical HMF, subject-specific HMF and sICA is obtained for each task in each of the ten sessions. To relate modes across individual sessions in the MSC data, an association matrix is obtained with CD-NMF as described in the ensemble averaging framework outlined in Fig. 2.

*Comparison of HMF modes to task timings.*

Canonical HMF is applied to MSC tasks and Brainomics task. Several combinations of task timings are compared to neural activation estimates of the HMF modes. In the motor task, task timings for all visual cues, all foot, all hand, left hand, right hand, left foot, right foot and tongue movement are compared to neural activation time courses of HMF modes. In incidental memory tasks as well as spatial and verbal discrimination task, task timings of the two visual cue types and their combination are compared to neural activation time course estimates. And last, in the Brainomics task, task timings are grouped into audio, video, vertical checkerboard, horizontal checkerboard, left hand, right hand, phrases and calculus and compared to neural activation time courses of HMF modes. The HMF mode with the highest correlation between task timing and neural activation is determined and in the following referred to as “the most task-relevant mode”.

*Comparison of subject-specific HMF with canonical HMF.*

HRF estimates are obtained by using a three basis function (canonical HRF + first and second derivative) GLM analysis. In the MSC cohort, voxel-wise GLM estimates are obtained with task timings of all visual stimuli cues from incidental memory tasks. In the Brainomics cohort, voxel-wise GLM estimates are obtained with video stimuli cue timings. In both data sets, a second level analysis on subject GLM estimates produces a spatial map, which is z-transformed and thresholded at 3 standard deviations. The thresholded spatial map is used to obtain an average HRF estimate per subject. The correlation of neural activation time course and task timings of the most task-relevant mode is compared between canonical and subject-specific HMF.

*Comparison of sICA with canonical HMF.*

In order to compare sICA with canonical HMF, task timing groupings are convolved with the canonical HRF to derive a generic task design. The ICs with the highest correlation between generic task design and time course of the IC are presented and compared to the most task-relevant mode of canonical HMF.

Additionally, the reproducibility of spatial map estimates of the most task-relevant IC and canonical HMF mode is compared across the ten sessions of the MSC data. The reproducibility of spatial maps is computed by the correlation between all pairs of spatial maps that are associated with the corresponding all-session average spatial map of the most task-relevant IC or canonical HMF mode.

## 4 Results

The most task-relevant modes were compared between canonical HMF, subject-specific HMF and sICA in motor, discrimination, incidental memory and Brainomics tasks. Firstly, the correlation between task timing groupings and neural activation time course was compared between canonical and subject-specific HMF. Secondly, the correlation between BOLD time course and task design of the corresponding task timing groupings was compared between canonical HMF and sICA. And lastly, the spatial reproducibility of the most task-relevant modes across the 10 sessions of the MSC cohort were compared between canonical HMF and sICA. Summary statistics of the average correlation are provided for individual stimulus types in each task.

### 4.1 Simulated data

**Fig. 4:**
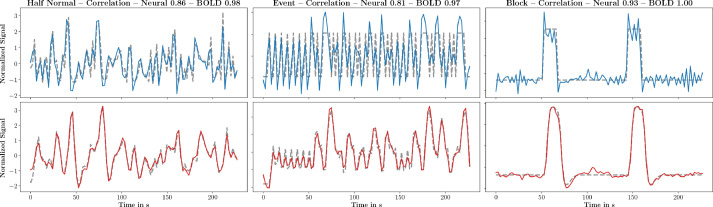
Original and estimated neural activation (top row) and corresponding BOLD time courses (bottom row) obtained by canonical HMF for random- (left), event- (middle) and block-type (right) neural activations. Estimated and original neural activation time courses were normalized to zero mean and unit standard deviation for presentation.

Canonical HMF and subject-specific HMF recovered all 14 simulated brain networks. The median correlation between original and estimated neural activation obtained with canonical HMF was 0.78, 0.76, and 0.95 for random, event and block type stimulus, respectively. The median correlation between original and estimated BOLD time courses was 0.98, 0.97, and 0.99 for random, event and block type stimulus, respectively. Discontinuous neural activation change in block- or event-type neural activation was less well recovered than continuous change in random neural activation by HMF as depicted in Fig. 4.

**Fig. 5:**
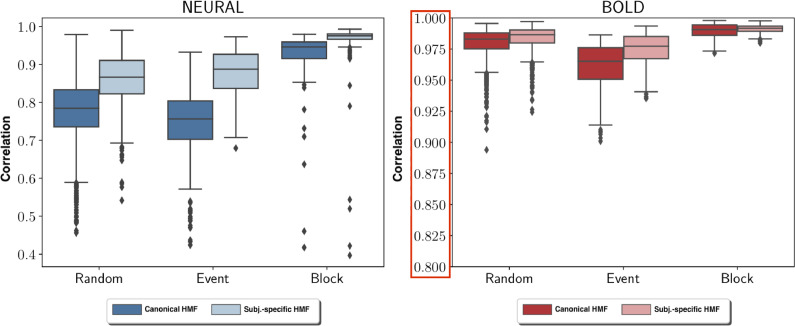
The distribution of correlation between original and estimated neural activation time courses (left) as well as the distribution of correlation between original and estimated BOLD time courses (right) for random, event and block type activation, respectively, using either canonical HMF or subject-specific HMF.

Using subject-specific HMF instead of canonical HRF, the median correlation between original and estimated neural activation increased to 0.87, 0.89, and 0.98 for random, event and block neural activation, respectively. The median correlation between original and estimated BOLD time courses increased to 0.99, 0.98, and 0.99 for random, event and block neural activation, respectively. The median correlation difference between canonical and subject-specific HMF was caused by a mismatch between canonical and actual HRF in each subject. This HRF mismatch translated into a mismatch between original and estimated neural activation, which resulted in low correlation depicted in Fig. 5 in subjects whose HRF largely deviated from the canonical HRF. The more mismatch there was in the time-to-peak delay between actual and canonical HRF, the lower the observed correlation between estimated and actual neural activation time course. In contrast, the effect on BOLD time course estimation was marginal. Only a small improvement in the correlation between original and estimated BOLD time courses was observed when comparing subject-specific with canonical HMF.

### 4.2 Imaging data

In the following , the alignment between canonical HMF modes and task time groupings in each individual task is examined. Then, modes from subject-specific HMF are estimated and compared to modes from canonical HMF with respect to their alignment to task timings. Finally, spatial maps and BOLD time courses of modes from canonical HMF are compared to corresponding ICs of sICA.

#### 4.2.1 HMF produces modes whose neural activation time course resembles task timings

In order to determine how well HMF recovers neural activation time courses in task scenarios ranging from block to fast random task designs, HMF was applied to tasks of the MSC and Brainomics cohort.

**Fig. 6:**
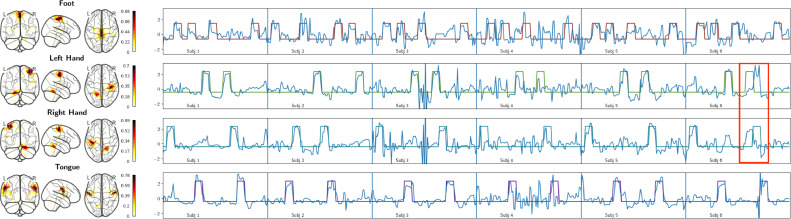
Each row depicts task blocks (lines in four distinct colors in row one to four) of either foot, left hand, right hand or tongue movement, respectively. The neural activation time course of the mode with the highest correlation to a particular movement type is depicted as continuous blue line superimposed on the individual block movement timings.

Regarding the MSC motor task, Fig. 6 shows the spatial maps and the neural activation time course obtained by canonical HMF on the motor task along with the timing blocks for the first six subjects of session one. More specifically, from top to bottom, each row depicts the following movements: foot, left hand, right hand, and tongue. The obtained neural activation followed individual task timings in subjects 1,2, and 5. On the other hand, in subjects 3, 4 and 6, the neural activation only partially followed the task timings. However, most importantly, HMF exposed that subject 6 confused moving left with right hand in one task block as highlighted by the red bounding box.

**Fig. 7:**
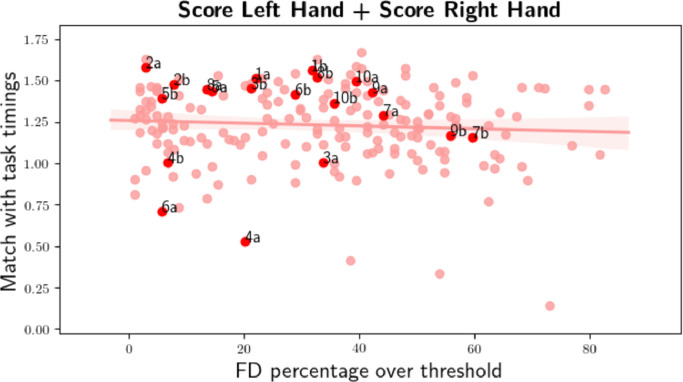
Total correlation score plotted against the percentage of time points over frame-wise displacement (FD) threshold in the MSC motor task. The labelled data points correspond to the first and second scan of session one of subject one to ten. The y-axis depicts TCS, while the x-axis depicts the percentage of time points that are over the frame-wise displacement threshold.

In order to find more scans in which the subject confuses instructions in the motor task, the correlation between task timings and neural activation time course of the most task-relevant mode is computed. Since for hand movement there are two correlation scores, a total correlation score (TCS) is defined as the sum of the correlations computed for the left and right hand task timing. Fig. 7 shows TCS computed for the first and second scan of all the subjects and all the sessions plotted against the percentage of time points over frame-wise displacement (FD) threshold in the MSC motor task. In the figure, only the points corresponding to the first session are labelled with na or nb where n=(1...10) indicates the subject, and a and b indicate the first and second scan, respectively.

Estimated neural activation time course and task timings showed high accordance in task runs 1a, 1b, 2a, 2b, 5a and 5b as seen in Fig. 6, which translated into the high correlation scores in Fig. 7. In contrast, task runs 3a, 4a and 6a had lower scores given their worse alignment with task timings. However, the second task runs 3b, 4b and 6b of these subjects had better alignment with task timings (similarly high correlation values as in subject 1, 2, and 5) with similarly experienced head motion. A small negative correlation (≈−0.1) was observed between FD and TCS.

Fig. 7 shows that if a subject had a low TCS value it happened only in one of the two scans. This suggests that HMF did not under-perform in specific subjects but subjects potentially did not comply with the given task. Moreover, scans of subjects with TCS below 0.75 provide evidence for non-compliant task behavior.

**Fig. 8:**
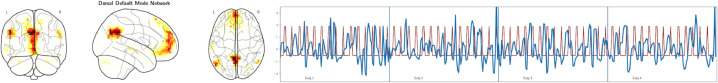
Spatial map and neural activation time course of the dDMN in the MSC motor task. The neural activation time course of task run 1 of the first four subjects (blue line) is superimposed on corresponding task timings of visual instruction cues (red line). The median correlation between visual cue timings and neural activation time course is −0.23.

In addition to task-relevant modes, HMF also produced several intrinsic brain activity modes including dorsal and ventral default mode network (dDMN and vDMN), precuneus network (PREC), left and right central executive network (LCEN and RCEN), visuospatial network (VSN), and anterior salience network (ASN). The dDMN presented with an antagonistic behavior to visual cues as, for example, depicted in Fig. 8 for task run 1 of subject 1 to 4 in the first session of the MSC motor task. The Supplementary Material contains additional information about obtained intrinsic activity modes and their relation to the motor task stimuli.

**Fig. 9:**
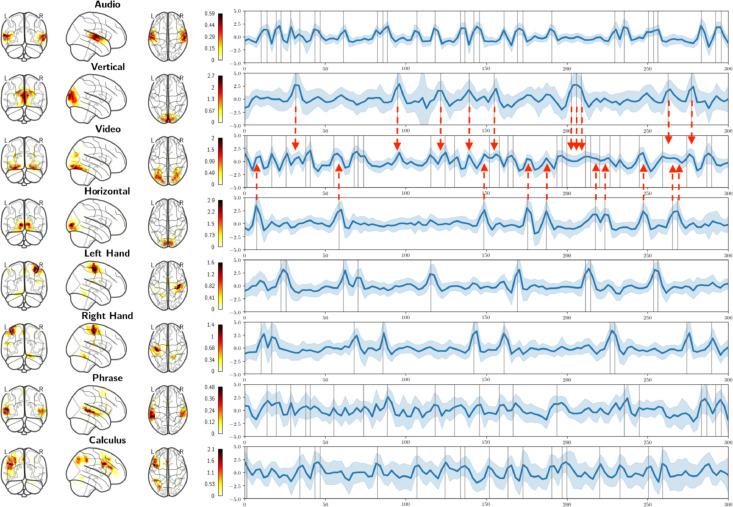
The mean (dark blue line) and standard deviation (light blue interval) of 90 Brainomics subjects of HMF modes whose neural activation time course correlates most with either left button click, right button click, auditory, visual, horizontal checkerboard, vertical checkerboard, calculus as well as sentence stimuli. Modes for vertical and horizontal checkerboard stimuli co-activate with the mode for video processing (red dashed errors).

In order to demonstrate that the proposed approach can recover neural activation in tasks with short stimulus presentation with the same hyper-parameter setting, HMF was also applied to the Brainomics data cohort. The neural activation times courses of the obtained modes followed visual, auditory, motor, and higher cognitive function processing stimuli. Fig. 9 depicts the spatial map and neural activation time courses of the obtained modes among the individual stimulus timings. Also for this cohort, neural activation time courses followed task timings of individual stimulus types. However, there was no exact one to one relationship in all modes between neural activation time course and task stimuli. One mode activated only for auditory stimuli (row 1) whereas the mode with the highest correlation to video stimuli (row 3) activated also for horizontal and vertical checkerboard stimuli (rows 2 and 4) as indicated by the dashed red arrows. The neural activation time courses of the mode associated with auditory stimuli had the lowest standard deviation across subjects followed by modes for video, horizontal checkerboard, left and right hand (rows 5 and 6), and vertical checkerboard stimuli. The HMF modes associated with all phrases or calculus stimuli (rows 7 and 8) showed the highest standard deviation in neural activation time courses across subjects.

#### 4.2.2 Subject-specific HMF outperforms canonical HMF in neural activation estimation

In order to determine if using subject-specific HRFs instead of the canonical HRF in HMF would result in modes whose neural activation time courses align better with task timings, subject-specific HRFs were first obtained with GLM analysis and replaced the canonical HRF in the Toeplitz matrix of HMF. The obtained HRF mean and standard deviation of all 10 subjects of the MSC data are depicted in Figure S1.

**Fig. 10:**
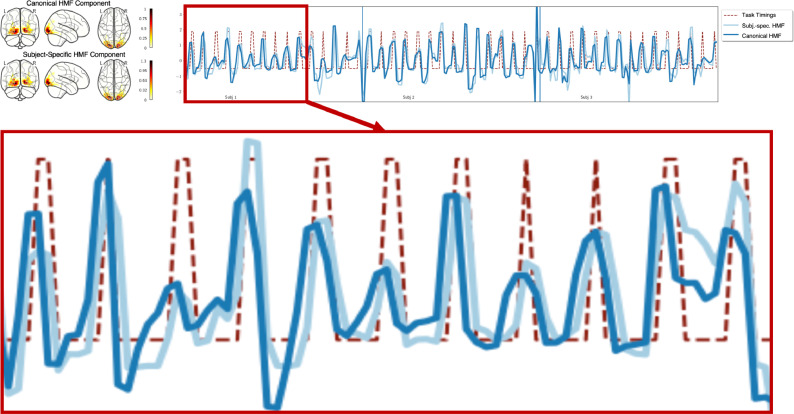
The neural activation time courses and spatial maps of the most correlated canonical and subject-specific HMF mode in motor task session one. The enlarged view of neural activation time courses showed that there is a small delay between the neural activation time course of canonical and subject-specific HMF. The canonical HRF takes a longer time to peak than the subject-specific HRF of subject 1 depicted in Fig. The neural activation time course of the corresponding HMF mode with canonical HRF therefore peaks earlier than the neural activation time course of the HMF mode with subject-specific HRF. Ultimately, the subject-specific HRF resulted in a better alignment with task timings (red dashed line) compared to the canonical HRF.

Fig. 10 depicts the difference in the neural activation estimate between the mode obtained with canonical and subject-specific HMF for the first three subjects of the MSC motor task in session one. Both modes correspond to areas of the visual cortex activated by visual cues presented during the motor task. The neural activation time course (light blue line) produced by subject-specific HMF had a better alignment to task timings (red dashed line) than the neural activation time course (dark blue line) produced by canonical HMF as highlighted in the increased view for subject one.

Table 1 shows median and median absolute deviation (MAD) of the most task-relevant mode for each stimulus type in MSC tasks and Brainomics task. Subject-specific HMF achieved a greater median correlation to the original task timing than canonical HMF in all MSC tasks, and a greater median correlation in six of eight task timings of the Brainomics task. Subject-specific HMF was statistically^4^ better than canonical HMF in ten out of nineteen of the examined task timings for both MSC and Brainomics tasks, while for nine task timings there was no statistical difference between the two approaches.

**Table 1:** Median ± MAD of the correlation between task timings and neural activation estimates for the most task-relevant mode for either canonical or subject-specific HMF. The best approach with respect to the median correlation is highlighted with bold font, the superscript ${}^{†}$ represents significantly better (*p-value*<0.05) correlation.

		Motor						Incidental Memory			Glass	
		Foot	Left Hand	Right Hand	Tongue	Block	Motor Cue	Faces	Scenes	Words	Spatial	Verbal
	Canonical HMF	0.36±0.18	0.63±0.14	0.65±0.16	0.68±0.1	0.32±0.13	0.27±0.1	0.41±0.11	0.45±0.08	0.38±0.13	0.42±0.1	0.26±0.1
	Subject-specific HMF	**0.48**±**0.19**${}^{†}$	**0.64**±**0.13**	**0.67**±**0.16**${}^{†}$	**0.7**±**0.08**${}^{†}$	**0.4**±**0.16**${}^{†}$	**0.31**±**0.13**	**0.46**±**0.13**	**0.51**±**0.07**${}^{†}$	**0.43**±**0.12**${}^{†}$	**0.5**±**0.09**${}^{†}$	**0.27**±**0.11**
		**Brainomics**										
		Audio	Video	Vertical	Horizontal	Left Hand	Right Hand	Phrase	Calculus			
	Canonical HMF	0.23±0.07	0.18±0.06	**0.22**±**0.08**	0.17±0.08	0.25±0.09	0.28±0.1	0.12±0.06	**0.15**±**0.08**${}^{†}$			
	Subject-specific HMF	**0.27**±**0.06**${}^{†}$	**0.21**±**0.07**${}^{†}$	0.21±0.08	**0.18**±**0.07**${}^{†}$	**0.29**±**0.09**	**0.3**±**0.08**	**0.13**±**0.07**	0.14±0.08			

#### 4.2.3 ICA produced more noisy mode estimates than HMF

Independent components (ICs) were obtained with sICA. Given that BOLD time course estimates of canonical and subject-specific HMF were highly similar, as shown in corresponding correlation distributions in Figures S6, S7 and S9.

**Fig. 11:**
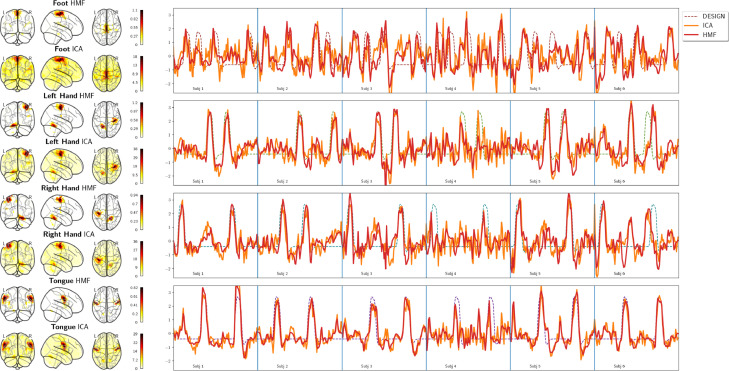
The juxtaposition of spatial maps and BOLD time courses of ICs and canonical HMF modes, which were the most task-relevant for foot, left and right hand, and tongue movements. The task design corresponds to the individual task timings in Fig. 6. The BOLD time courses correspond to neural activation time courses depicted in Fig. 6.

Fig. 11 shows spatial maps and BOLD time courses of modes found by canonical HMF and sICA for the first session of the MSC motor task. The confusion between right and left hand movements seen in the neural activation time course of subject six in Fig. 6 was also evident in the BOLD time course produced by canonical HMF and sICA as depicted in Fig. 11. As the figure shows, the modes found by sICA and canonical HMF had highly similar spatial maps and BOLD time courses, but modes produced by canonical HMF were spatially and temporally smoother than their sICA counterparts. The same holds also for the other MSC and Brainomics tasks as summarized in Tables 2 and 3.

**Table 2:** Median ± MAD of canonical HMF and sICA of the correlation between task timings convolved with canonical HRF and BOLD time course estimates. The best method with respect to the median correlation is highlighted with bold font, the superscript ${}^{†}$ represents significantly better results (*p-value*<0.05).

		Motor						Incidental Memory			Glass	
		Foot	Left Hand	Right Hand	Tongue	Block	Cues	Faces	Scenes	Words	Spatial	Verbal
BOLD	Canonical HMF	**0.51**±**0.21**${}^{†}$	**0.75**±**0.10**${}^{†}$	**0.78**±**0.14**${}^{†}$	**0.78**±**0.08**${}^{†}$	**0.44**±**0.16**${}^{†}$	**0.34**±**0.12**	**0.52**±**0.11**	0.54±0.09	**0.49**±**0.14**${}^{†}$	**0.46**±**0.12**${}^{†}$	**0.32**±**0.13**
	sICA	0.33±0.17	0.69±0.10	0.68±0.16	0.73±0.10	0.38±0.16	0.33±0.12	0.51±0.07	**0.57**±**0.06**	0.44±0.10	0.38±0.11	0.31±0.13
		**Brainomics**										
		Audio	Video	Vertical	Horizontal	Left Hand	Right Hand	Phrase	Calculus			
**BOLD**	Canonical HMF	**0.59**±**0.08**	**0.47**±**0.12**	**0.35**±**0.10**	**0.41**±**0.15**	0.41±0.20	0.38±0.09	**0.36**±**0.10**	**0.31**±**0.13**			
	sICA	0.58±0.08	0.40±0.11	0.32±0.09	0.37±0.11	**0.49**±**0.14**	**0.41**±**0.11**	0.33±0.11	0.30±0.15

**Table 3:** Median ± MAD of the reproducibility of the spatial maps obtained by canonical HMF and sICA. The best approach is in bold, the superscript ${}^{†}$ represents statistically significant better/worse results when compared to sICA (*p-value*<0.05).

		Motor						Incidental Memory			Glass	
		Foot	Left Hand	Right Hand	Tongue	Block	Cues	Faces	Scenes	Words	Spatial	Verbal
**Spatial Repr.**	Canonical HMF	**0.92**±**0.05**${}^{†}$	**0.95**±**0.07**${}^{†}$	**0.95**±**0.17**	**0.97**±**0.01**${}^{†}$	**0.81**±**0.17**${}^{†}$	**0.89**±**0.12**	**0.91**±**0.09**${}^{†}$	**0.94**±**0.03**${}^{†}$	**0.90**±**0.22**${}^{†}$	**0.92**±**0.12**${}^{†}$	**0.86**±**0.12**
	sICA	0.65±0.13	0.89±0.06	0.87±0.22	0.90±0.09	0.58±0.2	0.79±0.19	0.81±0.02	0.84±0.06	0.78±0.04	0.82±0.02	0.79±0.06

Table 2 summarizes median and MAD of correlation between task design and BOLD time course of the most task-relevant mode for each stimulus type in each MSC and Brainomics task. Canonical HMF showed higher correlation than sICA in seventeen out of nineteen task designs.

Regarding the spatial reproducibility, canonical HMF obtained higher values compared to sICA in all nineteen MSC task-related spatial maps as shown in Table 3. HMF produced modes characterized by statistically higher median values of spatial reproducibility compared to sICA in eight out of eleven tasks.

## 5 Discussion

This paper proposed a novel matrix factorization technique called HMF, which decomposes fMRI data into modes. Each mode is composed of a neural activation time course, a BOLD time course and a spatial map. Task timings were not provided to HMF but were retrospectively matched with neural activation time courses of estimated modes to evaluate how well these task timings were recovered.

For stimuli causing increased neural activation in the visual, auditory and motor areas of the eloquent cortex, HMF obtained modes whose neural activation time courses followed corresponding task timings with high correlation.

For the MSC motor task, HMF produced modes whose neural activation time courses corresponded with the experiment block design. Moreover, HMF also produced modes whose neural activation time courses followed sparse event-type task timings in MSC incidental memory, MSC spatial and verbal discrimination, and in the Brainomics task.

To further improve the correlation between neural activation time course and task timings in these modes, subject-specific HRF estimates were obtained by means of GLM analysis and used within HMF estimation. The obtained subject-specific HMF modes showed a statistically significant increase in correlation between neural activation estimates and task timings when compared to canonical HMF.

HMF tries to bridge the gap between hypothesis and data-driven analysis techniques in fMRI. The fMRI task experiment relies on two basic assumptions. Firstly, a task stimuli of certain duration causes an equally long period of increased neural activation in an on/off manner. Secondly, the translation of neural activation increase into BOLD response can be approximated by a hemodynamic forward model based on linear filter theory. Similarly to GLM analysis, HMF relies on both of these assumptions but, since it does not need task timings of the experiment to reveal involved areas in brain processing, it can be applied to a much wider range of tasks where these two assumptions might not hold, such as in intrinsic brain activity.

Moreover, HMF can reveal individual task scans where subjects did not comply with task instruction. This is a strong advantage over GLM analysis because unexpected subject behavior will lead to the derivation of wrong spatial activation maps. Tools such as HMF are therefore needed to retrospectively check task participation. For example, in circumstances in which task fMRI is currently in use for surgery planning (Karahanoğlu, Grouiller, Gaudes, Seeck, Vulliemoz, Van De Ville, 2013b, Lopes, Lina, Fahoum, Gotman, 2012), or in task experiments for newborns, which cannot be asked about their task performance or experienced intensity of a presented stimulus.

HMF is therefore proposed as a validation tool that enables reconstruction of task timings in fMRI task experiments. As shown in the experiments, inferred neural activation time courses matched task timings of foot, hand and tongue movements as well as visual cues in the MSC motor task. Accordingly, inferred neural activation time courses matched sparse task timings of auditory, video, horizontal and vertical checkerboard, and hand movements in the Brainomics task.

Furthermore, HMF provided insights of neural activation in task-free networks. For example, the dDMN was found to deactivate during visual stimuli presentation in the MSC motor task. HMF shares this ability with other BSS techniques, such as sICA, proposed to study brain networks in which ‘activation is difficult to predict‘ (McKeown et al., 1998). sICA (Beckmann and Smith, 2004) became one of the most prominent of such tools for analyzing task-unrelated brain networks in both task and resting-state fMRI experiments. Nonetheless, one of the problems of sICA is that it does not take into account the order of time points and spatial relationships of adjacent voxels. On the contrary, HMF leverages such intrinsic topological properties by using TV in the estimation process of spatial maps and time courses. Although obtained ICs are characterized by high spatial and temporal similarity with HMF modes, HMF produced modes with higher correlation with task design and greater spatial reproducibility when compared to sICA due to explicit spatial and temporal regularization.

Studies have shown intra-subject HRF variability to be lower than inter-subject variability (Aguirre, Zarahn, D’esposito, 1998, Handwerker, Gonzalez-Castillo, D’esposito, Bandettini, 2012). Therefore, subject-specific HRFs were chosen as an extension of HMF in this work, which resulted in a better alignment with task timings.

Other studies have hypothesized that the entire brain might respond to an extrinsic task by translating task stimulus into BOLD signal with varying HRFs across the brain (Gonzalez-Castillo et al., 2012). However, although the evidence for this assumption is low given the reported HRF magnitudes in Gonzalez-Castillo et al., 2012, varying HRFs across the cortex has not be conducted in this work but could be explored by having an individual Toeplitz matrix for each HMF mode.

Another limitation of the proposed approach is that the subject-specific HRFs used with HMF were predetermined with GLM analysis. The gradient of the cost function was derived with respect to neural activation time courses N. However, the gradient can also be derived with respect to Toeplitz matrix F. Constraining neural activation to piece-wise constant time courses such as proposed in Karahanoğlu et al. (2013a) or Cherkaoui et al. (2019) might enable an alternating or simultaneous derivation of neural activation time courses and hemodynamic filters.

Furthermore, hyper-parameters of HMF were optimized for an entire set of different task settings. Performance on individual tasks is expected to improve by task-specific hyper-parameter tuning, which will be explored in future work.

The proposed HMF approach is currently limited to spatial group maps. Nevertheless, many approaches have been proposed that include subject-specific spatial estimates (Harrison, Bijsterbosch, Segerdahl, Fitzgibbon, Duff, Smith, Woolrich, 2019, Harrison, Woolrich, Robinson, Glasser, Beckmann, Jenkinson, Smith, 2015, Varoquaux, Gramfort, Pedregosa, Michel, Thirion, 2011). Therefore, a possible future extension of the proposed HMF model could include subject-specific instead of spatial group maps.

Finally, task experiments that recruit higher cognitive function might evoke neural activation that strongly deviates from the simple assumptions of the hemodynamic forward model. Thus, one of the limitations of the proposed HMF model, shared with GLM analysis, is the assumption that the hemodynamic forward model holds for the entire cortex. However, the question to what degree higher cognitive function behaves in accordance with the hemodynamic forward model is beyond the scope of this paper and remains a challenging problem for future research.

## 6 Conclusion

A novel BSS technique called HMF has been proposed to obtain mode configurations of the brain while engaged in task processing. HMF recovered modes whose neural activation time course correlated well with corresponding stimulus timings in each task as shown in retrospective analysis in this work. Using the same hemodynamic forward model, HMF also recovered modes whose neural activation time courses reflected ongoing intrinsic neural activation during task processing. These modes were on average anti-correlated with task timings. fMRI research in the last decades has either focused on estimating the spatial extent of areas that are involved in task processing with GLM analysis or studied intrinsic brain activity of brain networks with BSS techniques such as sICA. Recent years have shown that this division is closing and that a novel class of techniques emerges that models fMRI data in its entirety by combing analysis of networks engaged in task processing and networks of ongoing intrinsic activity. The proposed HMF model is a step towards unification of resting-state and task fMRI analysis.

## CRediT authorship contribution statement

**Michael Hütel:** Conceptualization, Methodology, Data curation, Formal analysis, Writing - original draft, Visualization, Investigation. **Michela Antonelli:** Supervision, Writing - review & editing. **Andrew Melbourne:** Supervision. **Sebastien Ourselin:** Supervision.

## Appendix A

### A1 Approximation of spatial total variation

In order to compute spatial total variation with efficient matrix multiplications, the product of $H=\sigma ({\left(\mathrm{FN}\right)}^{⊤}Y+b)$ is rewritten element-wise as

(8) $$\left[\begin{array}{cccc} \sigma ({\left(\mathrm{FN}\right)}^{⊤}y_{1}+b) & \sigma ({\left(\mathrm{FN}\right)}^{⊤}y_{2}+b) & \ldots & \sigma ({\left(\mathrm{FN}\right)}^{⊤}y_{V}+b) \end{array}\right],$$

where $\sigma ({\left(\mathrm{FN}\right)}^{⊤}y_{v})$ constitutes a column vector for voxel v. All column vectors taken together form the spatial maps H as depicted in Eq. (8). The original data Y is rearranged into individual matrices along either dimension V1, V2 or V3 of the three-dimensional Euclidean space. An approximation of Eq. (3) is computed with the following term

(9) $$\begin{array}{cc} R_{H_{TV}}\;\;=\;\;\;\; & \beta_{1}\sum_{i}^{V2}\sum_{j}^{V3}{∥\sigma \left({\left(\mathrm{FN}\right)}^{⊤}Y_{\mathrm{ij}}^{\left(1\right)}+b\right)D_{1}^{⊤}∥}_{1}+\\ & \sum_{i}^{V1}\sum_{j}^{V3}∥\sigma \left({\left(\mathrm{FN}\right)}^{⊤}Y_{\mathrm{ij}}^{\left(2\right)}+b\right)D_{2}^{⊤}{∥}_{1}+\sum_{i}^{V1}\sum_{j}^{V2}{∥\sigma \left({\left(\mathrm{FN}\right)}^{⊤}Y_{\mathrm{ij}}^{\left(3\right)}\;+\;b\right)D_{3}^{⊤}∥}_{1} \end{array}$$

where $Y_{\mathrm{ij}}^{\left(1\right)}\in R^{T\times V1},$ $Y_{\mathrm{ij}}^{\left(2\right)}\in R^{T\times V2},$ $Y_{\mathrm{ij}}^{\left(3\right)}\in R^{T\times V3},$ $D_{1}\in R^{(V1-1)\times V1},$ $D_{2}\in R^{(V2-1)\times V2}$ and $D_{3}\in R^{(V3-1)\times V3}$. An example of the difference operator matrix $D_{i}$ is given in Eq. (10).

(10) $$D_{i}=\left(\begin{array}{cccccc} -1 & 1 & & & & \\ & -1 & 1 & & & \\ & & -1 & 1 & & \\ & & & \ddots & \\ & & & -1 & 1\\ & & & & -1 & 1 \end{array}\right)$$

### A2 Exponential distribution

The exponential distribution is given by:

(11) $$p_{\lambda }\left(x\right)=\left\{ \begin{array}{cc} \lambda e^{-\lambda x} & x\geq 0\\ 0 & x<0 \end{array}\right.$$

The Kullback–Leibler divergence between the desired rate parameter λ and the estimated rate parameter ${\hat{\lambda }}_{c}$ in mode c is derived as follows:

(12) $$\begin{array}{cc} \Delta \left(\lambda ∥{\hat{\lambda }}_{c}\right) & =E_{\lambda }\left(\log\frac{p_{\lambda }\left(x\right)}{p_{{\hat{\lambda }}_{c}}\left(x\right)}\right)\\ & =E_{\lambda }\left(\log\frac{\lambda e^{-\lambda x}}{{\hat{\lambda }}_{c}e^{-{\hat{\lambda }}_{c}x}}\right)\\ & =\log\left(\lambda \right)-\log\left({\hat{\lambda }}_{c}\right)-\left(\lambda -{\hat{\lambda }}_{c}\right)E_{\lambda }\left(x\right)\\ & =\log\left(\lambda \right)-\log\left({\hat{\lambda }}_{c}\right)+\frac{{\hat{\lambda }}_{c}}{\lambda }-1 \end{array}$$

The maximum likelihood estimate for the rate parameter ${\hat{\lambda }}_{c}$ of mode c is:

(13) $${\hat{\lambda }}_{c}=\frac{1}{\bar{x}}=\frac{n}{\sum_{i}x_{i}}$$

### A3 Gradients

(14) $$\begin{array}{cc} \frac{\partial J}{\partial N}= & F^{⊤}\left(M⊙\left(\mathrm{FN}\sigma \left({\left(\mathrm{FN}\right)}^{⊤}Y+b\right)-Y\right)⊙M\right)\sigma \left(Y^{⊤}\mathrm{FN}+b^{⊤}\right)+\\ & F^{⊤}Y\left(\left(\left(M^{⊤}⊙\left(\sigma \left(Y^{⊤}\mathrm{FN}+b^{⊤}\right)N^{⊤}F^{⊤}-Y^{⊤}\right)⊙M^{⊤}\right)\mathrm{FN}\right)⊙\sigma \left(Y^{⊤}\mathrm{FN}+b^{⊤}\right)\right) \end{array}$$

(15) $$\begin{array}{cc} \frac{\partial R_{tv}}{\partial N}= & \sum_{i}^{V2}\sum_{j}^{V3}Y_{\mathrm{ij}}^{\left(1\right)}\left(\left(D_{1}sign\left(D_{1}^{⊤}\sigma {\left({\left(\mathrm{FN}\right)}^{⊤}Y_{\mathrm{ij}}^{\left(1\right)}+b\right)}^{⊤}\right)⊙\sigma^{\prime }{\left({\left(\mathrm{FN}\right)}^{⊤}Y_{\mathrm{ij}}^{\left(1\right)}+b\right)}^{⊤}\right)\right)+\\ & \sum_{i}^{V1}\sum_{j}^{V3}Y_{\mathrm{ij}}^{\left(2\right)}\left(\left(D_{2}sign\left(D_{2}^{⊤}\sigma {\left({\left(\mathrm{FN}\right)}^{⊤}Y_{\mathrm{ij}}^{\left(2\right)}+b\right)}^{⊤}\right)⊙\sigma^{\prime }{\left({\left(\mathrm{FN}\right)}^{⊤}Y_{\mathrm{ij}}^{\left(2\right)}+b\right)}^{⊤}\right)\right)+\\ & \sum_{i}^{V1}\sum_{j}^{V2}Y_{\mathrm{ij}}^{\left(3\right)}\left(\left(D_{3}sign\left(D_{3}^{⊤}\sigma {\left({\left(\mathrm{FN}\right)}^{⊤}Y_{\mathrm{ij}}^{\left(3\right)}+b\right)}^{⊤}\right)⊙\sigma^{\prime }{\left({\left(\mathrm{FN}\right)}^{⊤}Y_{\mathrm{ij}}^{\left(3\right)}+b\right)}^{⊤}\right)\right) \end{array}$$

(16) $$\frac{\partial R_{N}}{\partial N}=sign\left(ND_{4}\right)D_{4}^{⊤}$$

(17) $$\frac{\partial R_{sparse}}{\partial N}=Ysign\left(\sigma {\left({\left(\mathrm{FN}\right)}^{⊤}Y+b\right)}^{⊤}\right)⊙\sigma^{\prime }{\left({\left(\mathrm{FN}\right)}^{⊤}Y+b\right)}^{⊤}.$$

## Supplementary materials

Supplementary Data S1 Supplementary Raw Research Data. This is open data under the CC BY license http://creativecommons.org/licenses/by/4.0/ Supplementary Data S1

## Footnotes

1 https://openfmri.org/dataset/ds000224/

2 http://brainomics.cea.fr/localizer

3 The code for both approaches is available at https://github.com/meinternational/HMF

4 The superscript ${}^{†}$ indicates significantly better (*p-value*<0.05) as obtained with a non-parametric Wilcoxon rank test between paired samples of the most task-relevant mode of subject-specific HMF and canonical HMF.
