# Supplementary material for: Hemodynamic matrix factorization for functional magnetic resonance imaging
Source: Neuroimage. 2021 May 1;231:117814. doi: 10.1016/j.neuroimage.2021.117814 (PMC8210649; doi:10.1016/j.neuroimage.2021.117814)
Supplement: Supplementary Data S1 — Supplementary Raw Research Data. This is open data under the CC BY license http://creativecommons.org/licenses/by/4.0/ [file mmc1.pdf]

Supplementary Material

Average estimated HRF per MSC subject

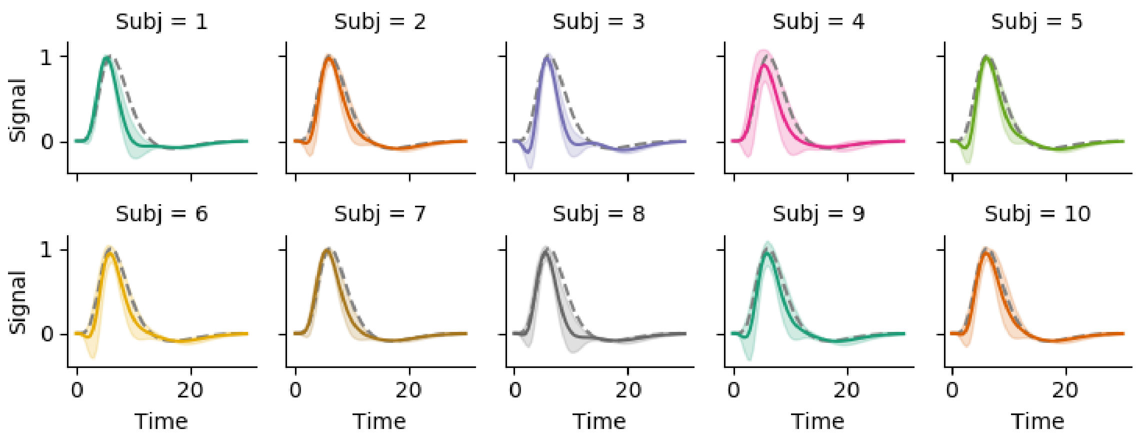

Figure S1: The average HRF (mean and standard deviation) for subject 1-10 of the MSC cohort estimated from the incidental memory tasks.

Example of modes for discrimination and incidental memory tasks

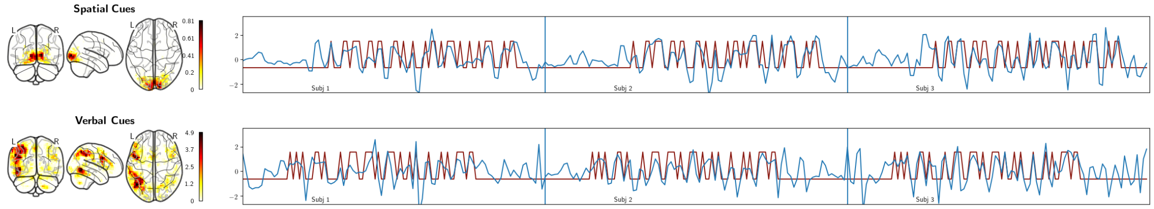

Figure S2: The task timings of coherent and incoherent stimuli combined (dark red line) for the spatial discrimination task (first row) and noun and verbal stimuli for the verbal discrimination task (second row). The spatial map and neural activation time course estimate (blue line) of the most task-relevant mode for either spatial disc. (first row) and verbal disc. (second row) task obtained with canonical HMF.

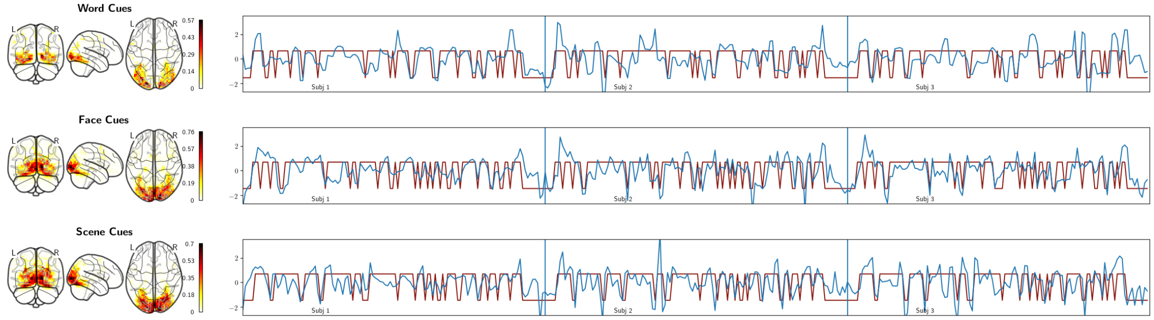

Figure S3: All task stimuli combined for either word (first row), face (second row) and scene incidental memory task (third row). Spatial map and neural activation time course estimate of the most task-relevant mode for the respective incidental memory task obtained with canonical HMF.

Spatial map of the mode most-correlated to the examined task timings for each session of the MSC cohort

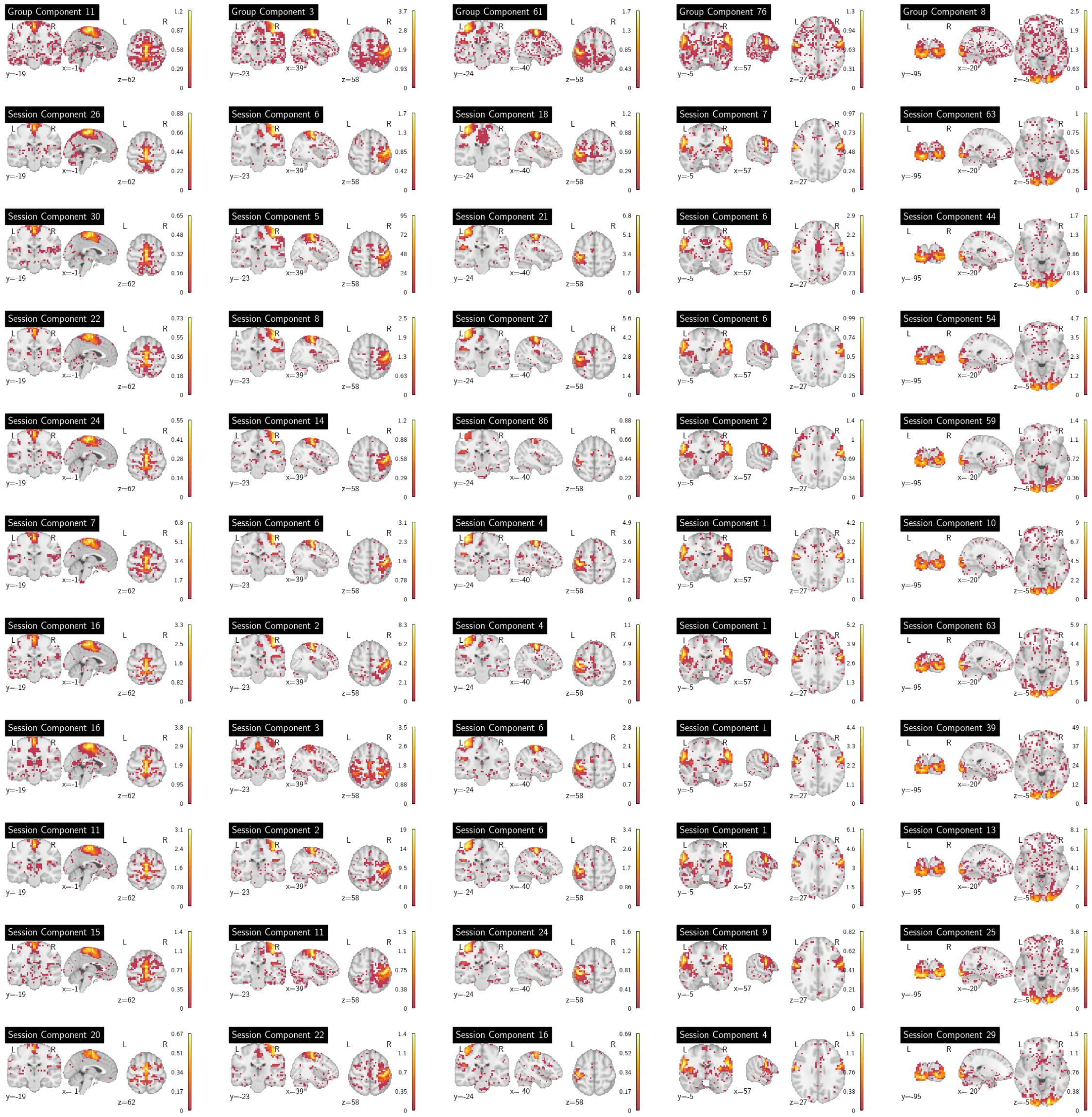

Figure S4: The first row contains the average spatial map of spatial maps of corresponding modes for session 1-10 (second to last row) for foot, left hand, right hand, tongue movement, and motor cues (left to right column). All modes were found among 40 modes in a canonical HMF decomposition in each session.

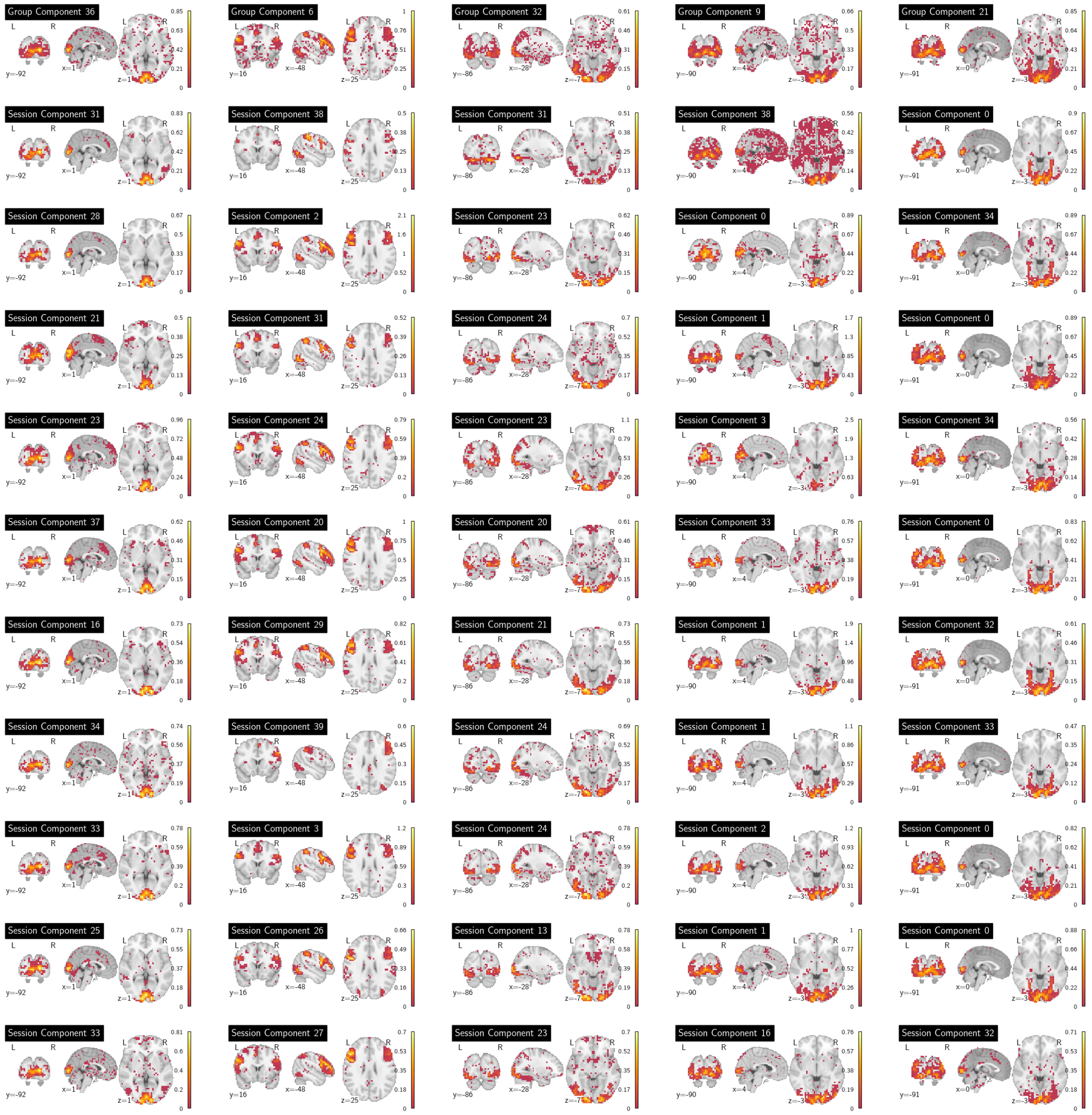

Figure S5: The first row contains the average spatial map of spatial maps of corresponding modes for session 1-10 (second to last row) for spatial and verbal discrimination task stimuli and incidental memory stimuli (words, faces, scenes). All modes were found among 40 modes in a canonical HMF decomposition in each session.

Correlation distributions for neural activation, BOLD and spatial estimates

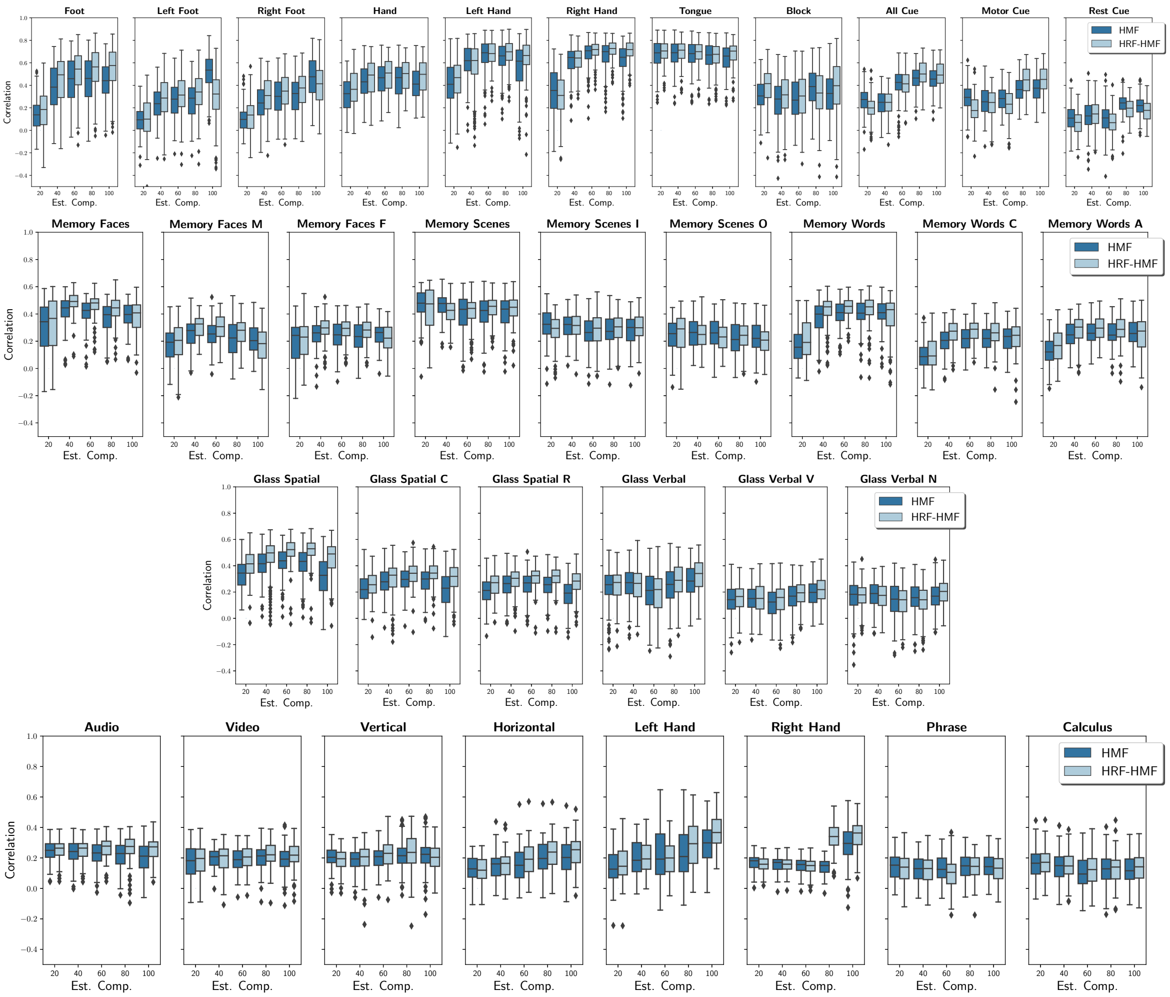

Figure S6: Correlation between task timing and neural activation estimate of the most task-relevant mode for canonical and subject-specific HMF dependent on the number of estimated modes. Motor task (1st row), incidental memory task (2nd row), glasslexical task (3rd row) and Brainomics task (4th row).

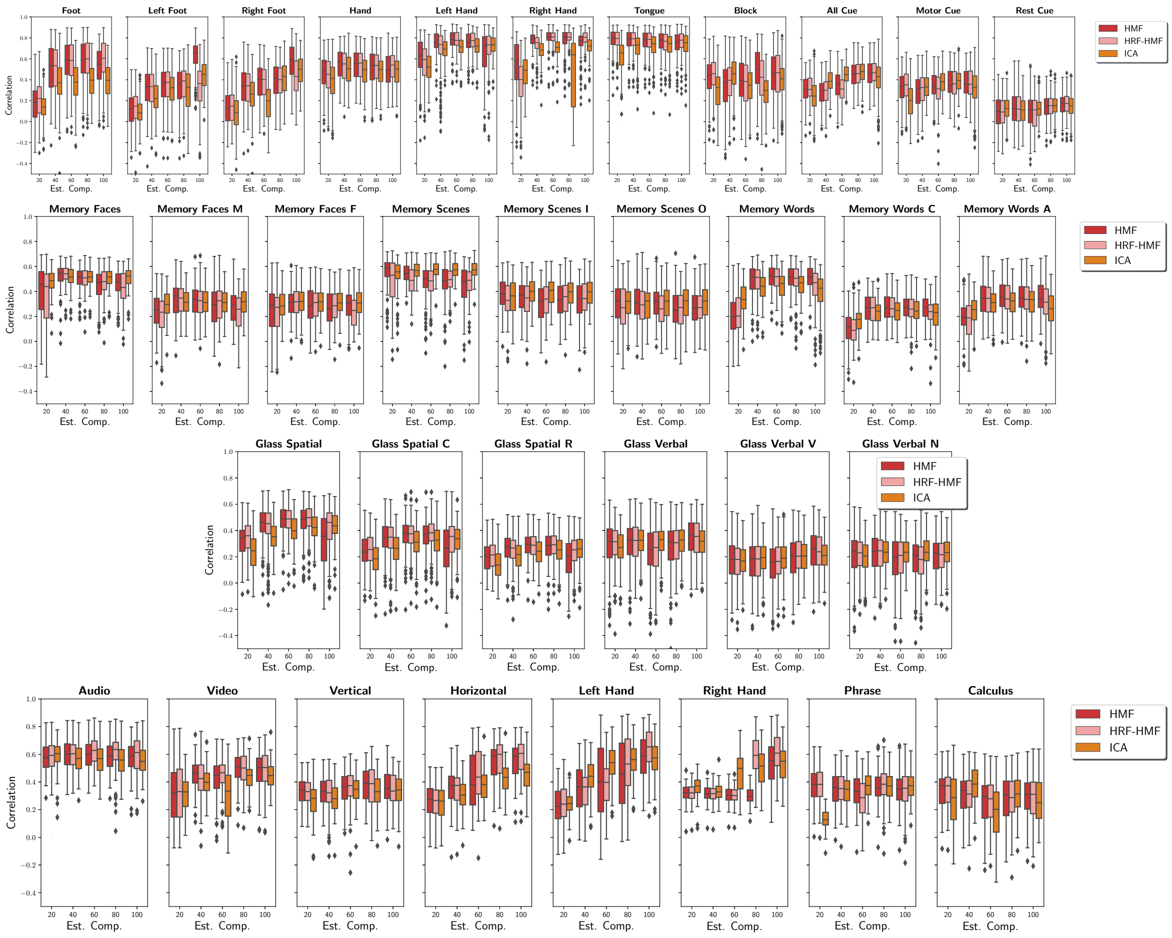

Figure S7: Correlation between task design and BOLD time course estimate of the most task-relevant mode for canonical HMF, subject-specific HMF and MELODIC dependent on the number of estimated modes. Motor task (1st row), incidental memory task (2nd row), glasslexical task (3rd row) and Brainomics task (4th row).

Figure S8

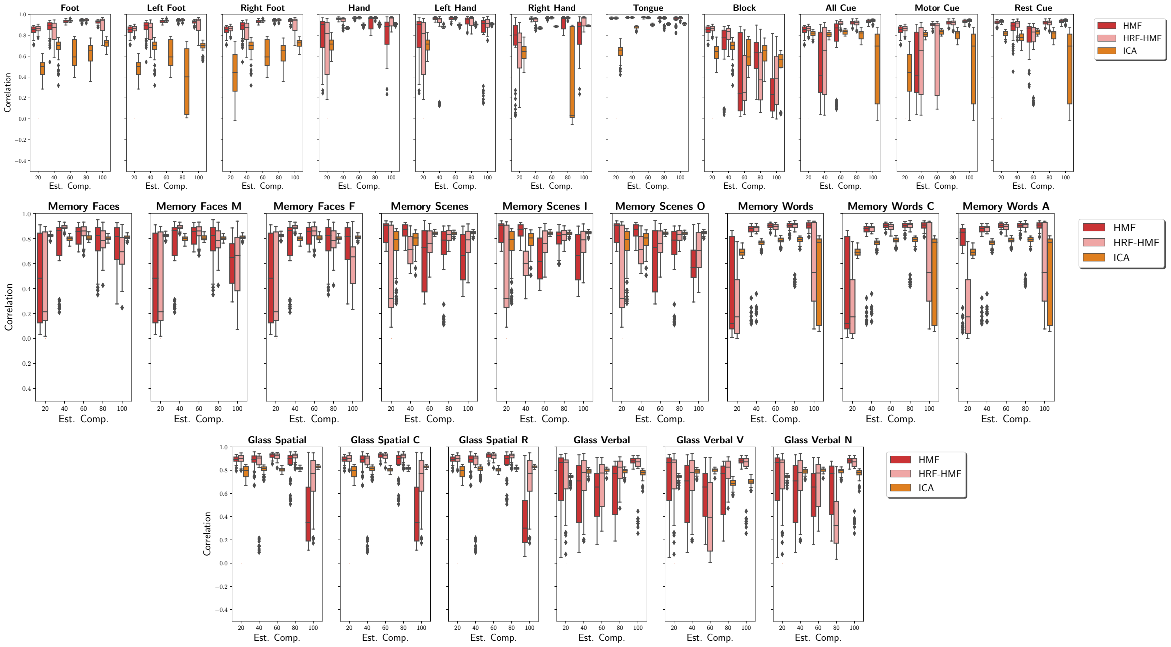

Figure S9: Spatial reproducibility among 10 sessions of the most task-relevant mode for canonical HMF, subject-specific HMF and MELODIC dependent on the number of estimated modes. Motor task (1st row), incidental memory task (2nd row) and glasslexical task (3rd row).

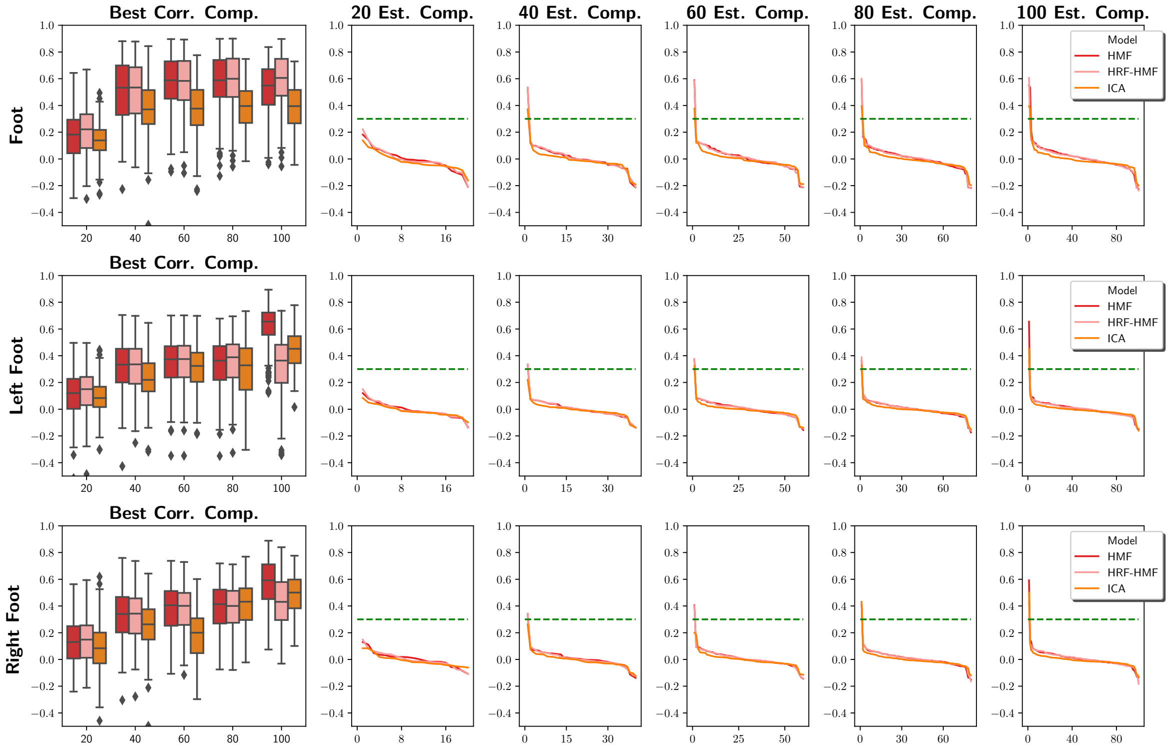

Figure S10: Correlation between task design and BOLD time course estimate of the most task-relevant mode obtained with canonical HMF, subject-specific HMF and MELODIC (first column). Median correlation between task design and BOLD time course estimate sorted in descending order for all modes of a decomposition in either 20 (second column), 40 (third column), 60 (fourth column), 80 (fifth column) and 100 modes (sixth column). The dotted green line marks a threshold of 0.3.

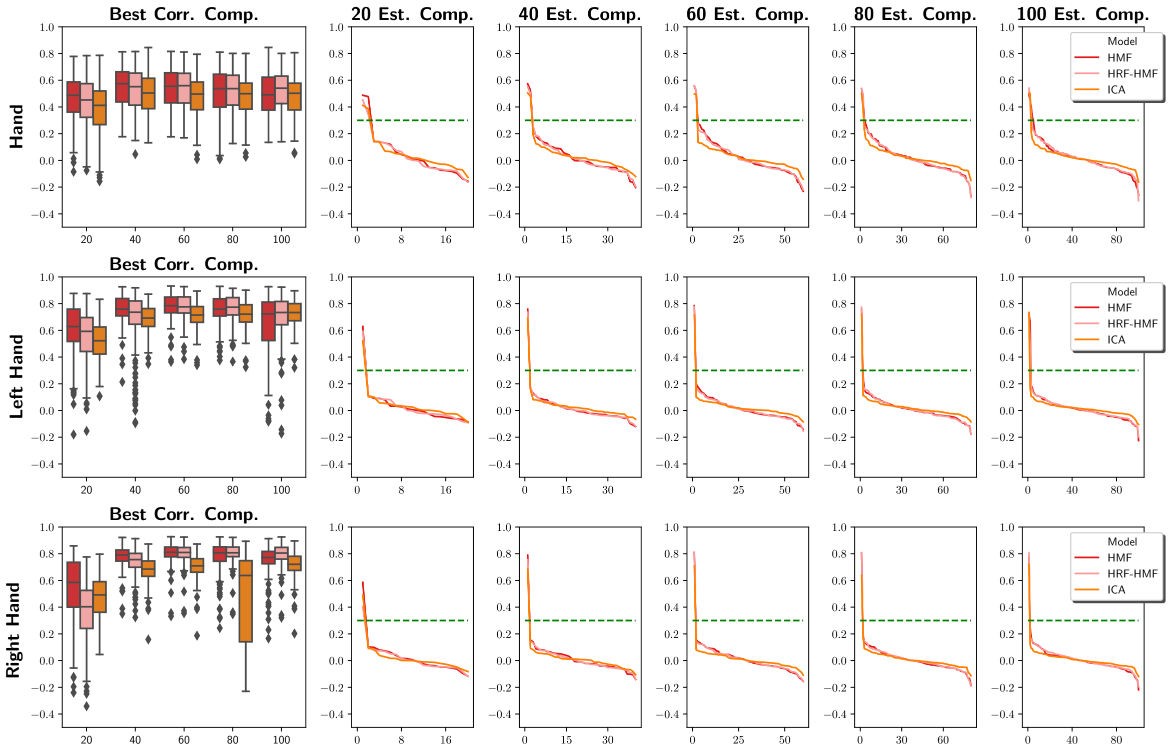

Figure S11: Correlation between task design and BOLD time course estimate of the most task-relevant mode obtained with canonical HMF, subject-specific HMF and MELODIC (first column). Median correlation between task design and BOLD time course estimate sorted in descending order for all modes of a decomposition in either 20 (second column), 40 (third column), 60 (fourth column), 80 (fifth column) and 100 modes (sixth column). The dotted green line marks a threshold of 0.3.

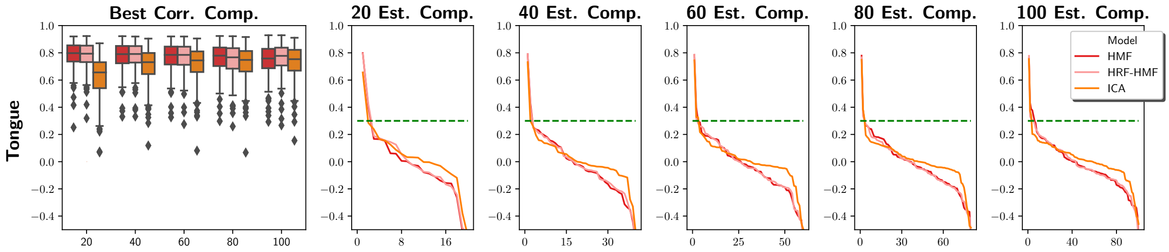

Figure S12: Correlation between task design and BOLD time course estimate of the most task-relevant mode obtained with canonical HMF, subject-specific HMF and MELODIC (first column). Median correlation between task design and BOLD time course estimate sorted in descending order for all modes of a decomposition in either 20 (second column), 40 (third column), 60 (fourth column), 80 (fifth column) and 100 modes (sixth column). The dotted green line marks a threshold of 0.3.

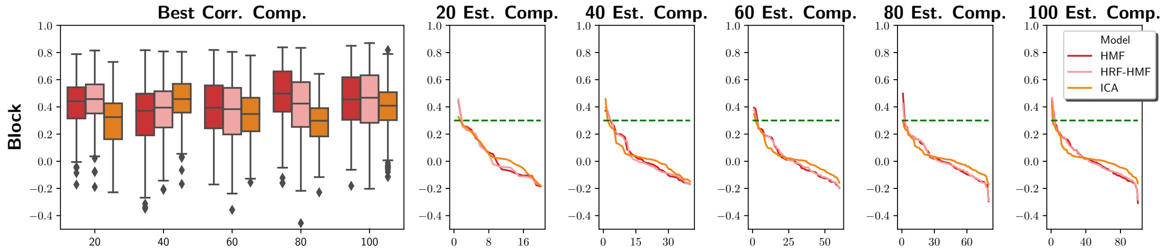

Figure S13: Correlation between task design and BOLD time course estimate of the most task-relevant mode obtained with canonical HMF, subject-specific HMF and MELODIC (first column). Median correlation between task design and BOLD time course estimate sorted in descending order for all modes of a decomposition in either 20 (second column), 40 (third column), 60 (fourth column), 80 (fifth column) and 100 modes (sixth column). The dotted green line marks a threshold of 0.3.

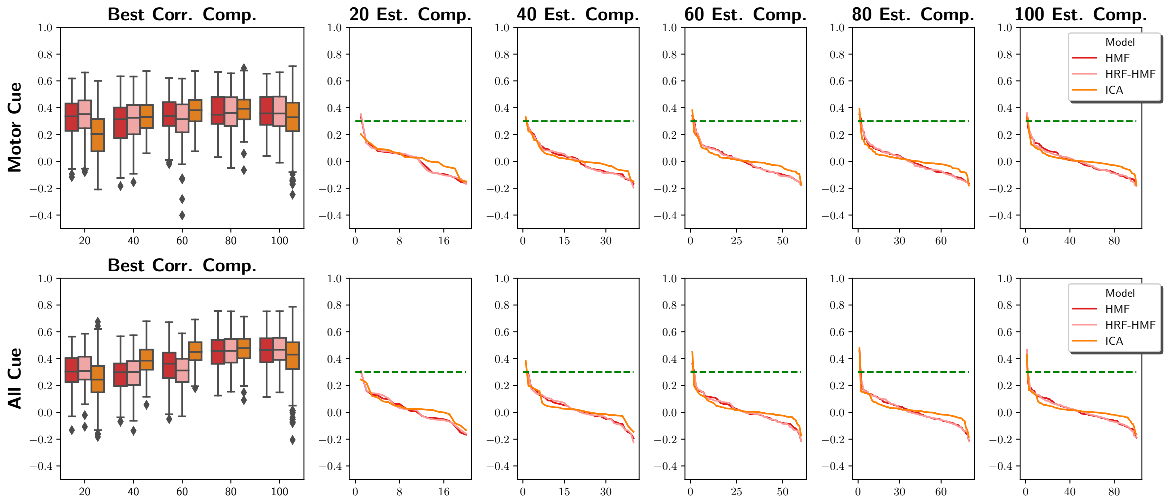

Figure S14: Correlation between task design and BOLD time course estimate of the most task-relevant mode obtained with canonical HMF, subject-specific HMF and MELODIC (first column). Median correlation between task design and BOLD time course estimate sorted in descending order for all modes of a decomposition in either 20 (second column), 40 (third column), 60 (fourth column), 80 (fifth column) and 100 modes (sixth column). The dotted green line marks a threshold of 0.3.

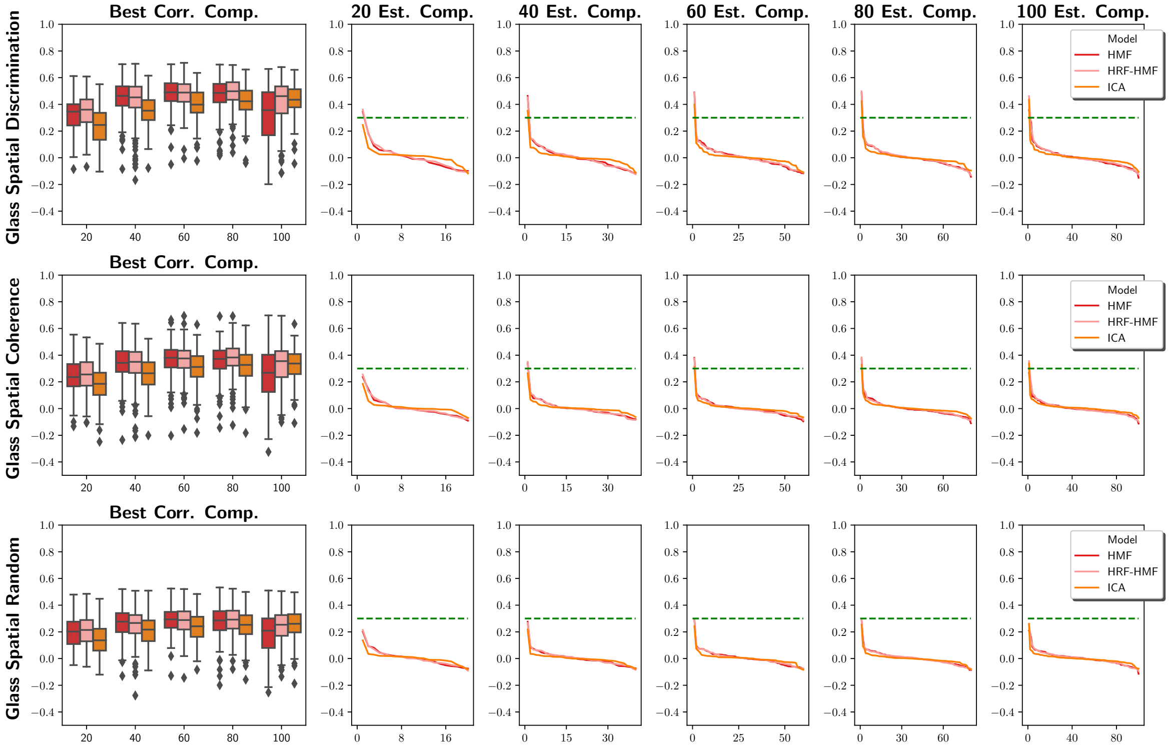

Figure S15: Correlation between task design and BOLD time course estimate of the most task-relevant mode obtained with canonical HMF, subject-specific HMF and MELODIC (first column). Median correlation between task design and BOLD time course estimate sorted in descending order for all modes of a decomposition in either 20 (second column), 40 (third column), 60 (fourth column), 80 (fifth column) and 100 modes (sixth column). The dotted green line marks a threshold of 0.3.

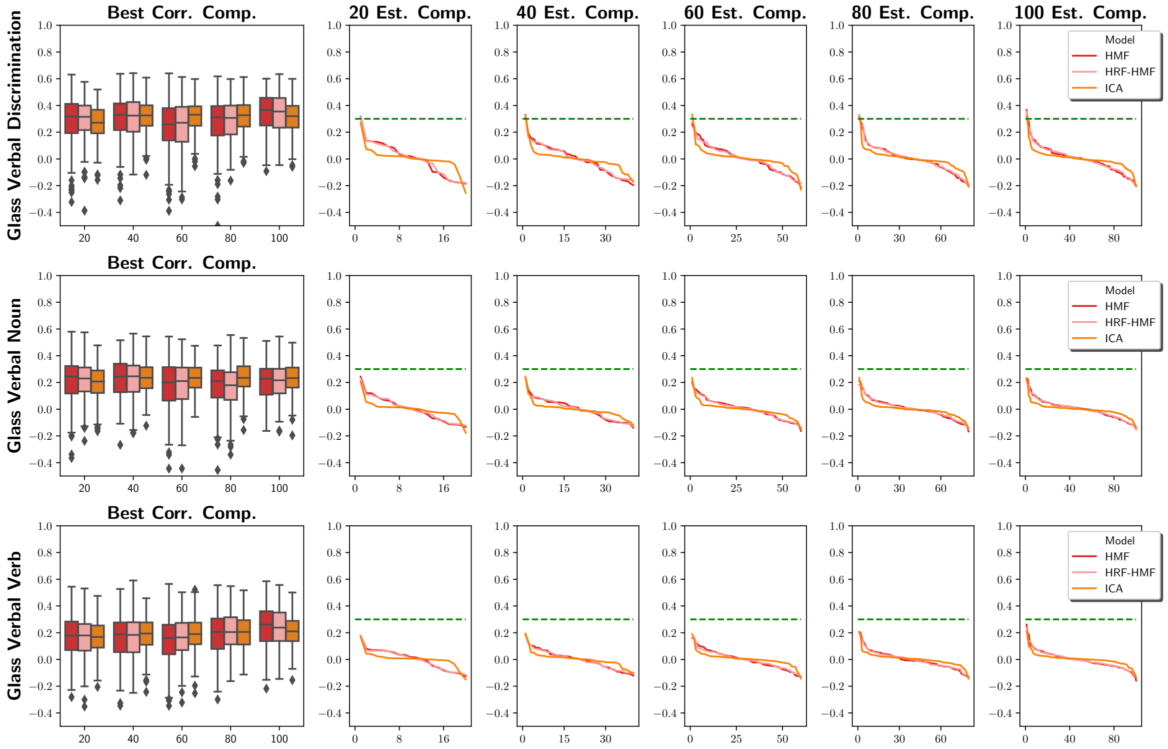

Figure S16: Correlation between task design and BOLD time course estimate of the most task-relevant mode obtained with canonical HMF, subject-specific HMF and MELODIC (first column). Median correlation between task design and BOLD time course estimate sorted in descending order for all modes of a decomposition in either 20 (second column), 40 (third column), 60 (fourth column), 80 (fifth column) and 100 modes (sixth column). The dotted green line marks a threshold of 0.3.

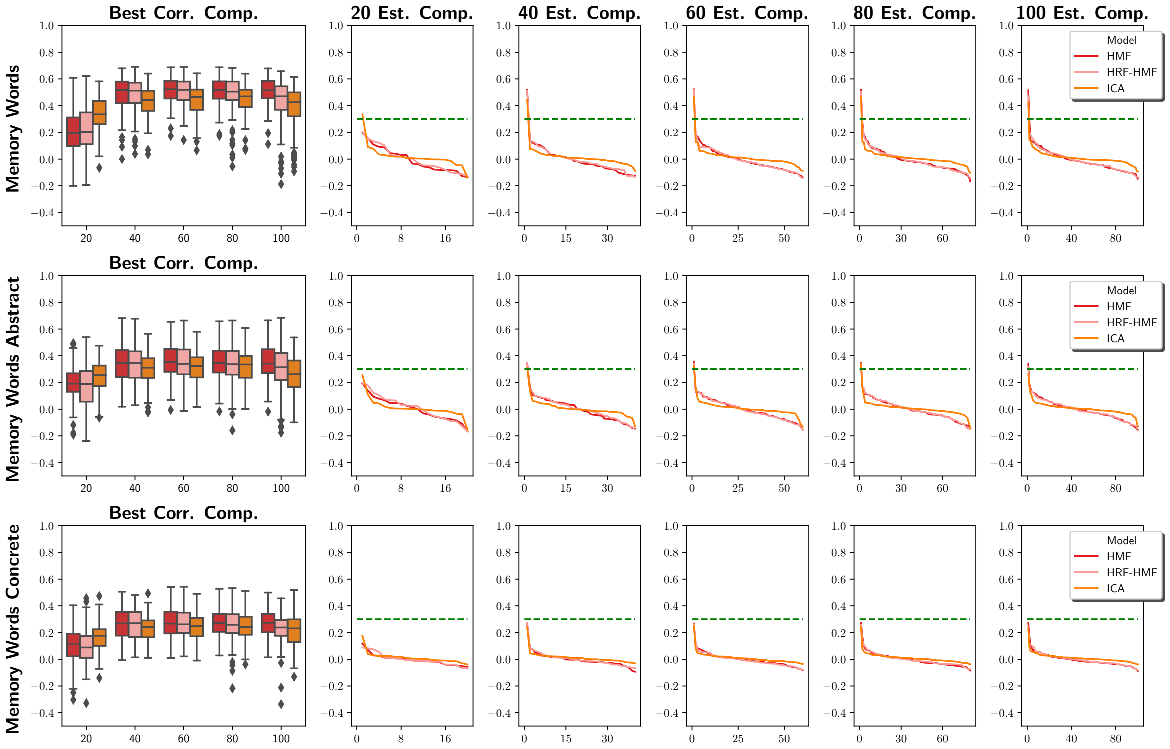

Figure S17: Correlation between task design and BOLD time course estimate of the most task-relevant mode obtained with canonical HMF, subject-specific HMF and MELODIC (first column). Median correlation between task design and BOLD time course estimate sorted in descending order for all modes of a decomposition in either 20 (second column), 40 (third column), 60 (fourth column), 80 (fifth column) and 100 modes (sixth column). The dotted green line marks a threshold of 0.3.

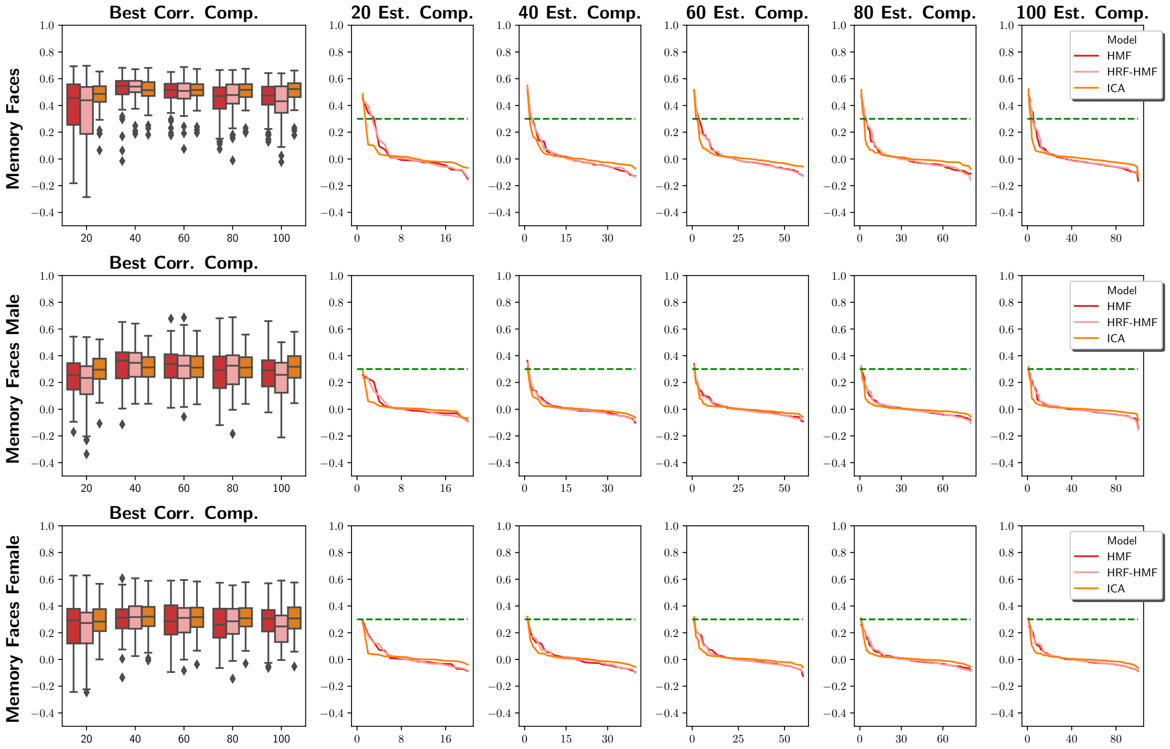

Figure S18: Correlation between task design and BOLD time course estimate of the most task-relevant mode obtained with canonical HMF, subject-specific HMF and MELODIC (first column). Median correlation between task design and BOLD time course estimate sorted in descending order for all modes of a decomposition in either 20 (second column), 40 (third column), 60 (fourth column), 80 (fifth column) and 100 modes (sixth column). The dotted green line marks a threshold of 0.3.

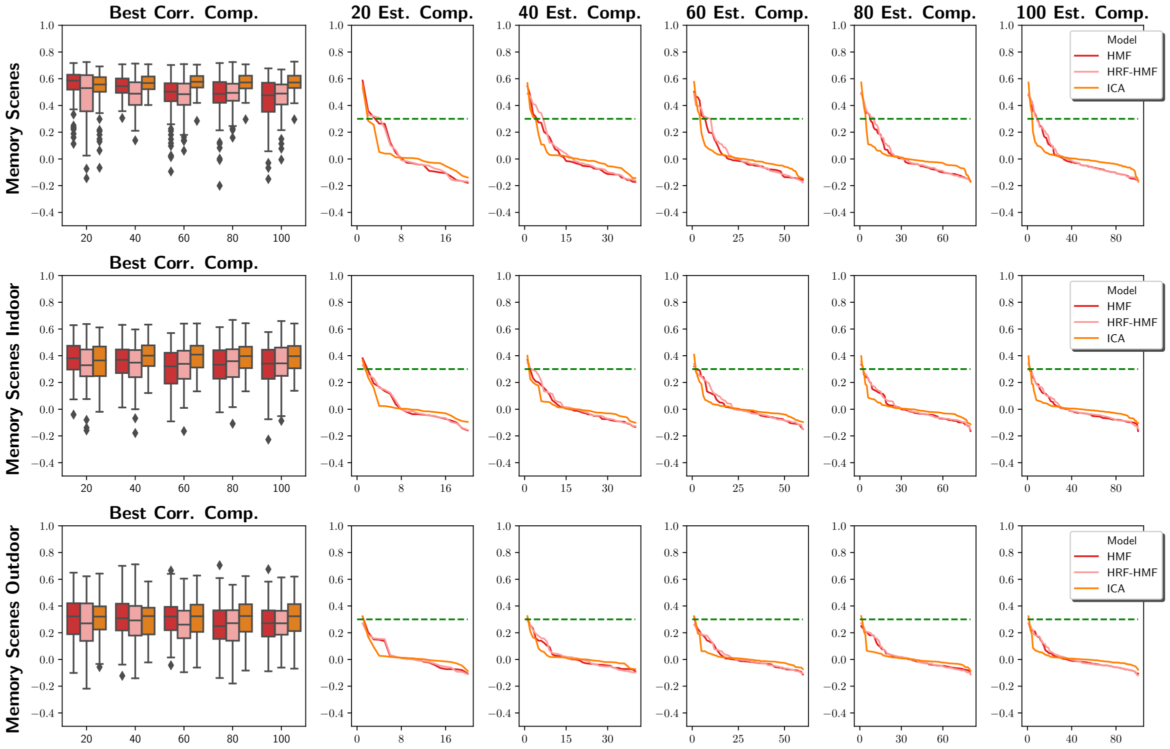

Figure S19: Correlation between task design and BOLD time course estimate of the most task-relevant mode obtained with canonical HMF, subject-specific HMF and MELODIC (first column). Median correlation between task design and BOLD time course estimate sorted in descending order for all modes of a decomposition in either 20 (second column), 40 (third column), 60 (fourth column), 80 (fifth column) and 100 modes (sixth column). The dotted green line marks a threshold of 0.3.

Brainomics GLM contrasts

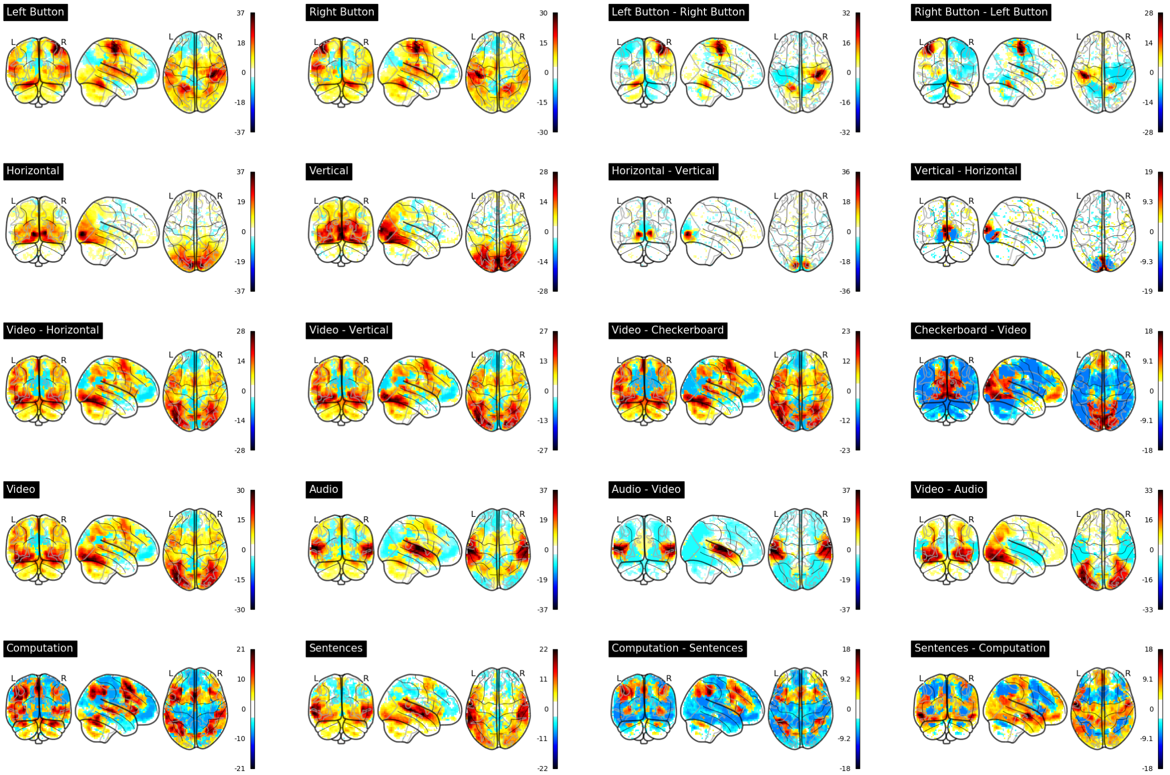

Figure S20: The spatial maps (z-values) of 20 computed contrasts obtained with a second level GLM analysis on the Brainomics task data.

Neural activation patterns in task-unrelated modes

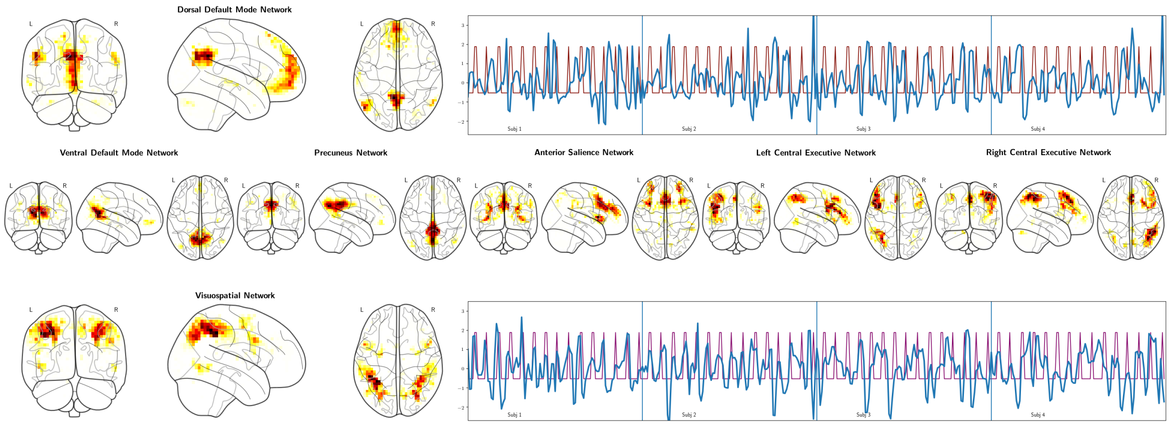

Figure S21: The first and third row depict spatial map and neural activation time course of modes that resembled the dorsal dDMN and VSN, respectively. The neural activation time course (blue line) is superimposed on task timings of the visual motor task cues (red line). The median correlation between visual cue task timings and estimated neural activation time course is -0.23 and 0.28, for dDMN and VSN, respectively (averaged over all sessions and subjects). The second row depicts vDMN, PN, ANS, LCEN, RCEN. The corresponding median correlations for these modes are -0.02, -0.07, 0.06, 0.08 and 0.24, respectively.

In contrast to HMF, the GLM enables to juxtapose distinct stimuli types (Fig. S20) but does not allow modeling of signal variations from so called resting-state or task-free brain networks. In the following, neural activation patterns in task-unrelated networks that presented during task performance were examined. Commonly known resting-state networks such as dorsal and ventral default mode network (dDMN and vDMN), Precuneus network (PREC), left and right central executive network (LCEN and RCEN), visuospatial network (VSN) and anterior salience network (ASN) were selected for detailed examination.

The seven corresponding modes of the MSC motor task of session one (among 40 estimated modes) are depicted in Figure S21. The first row depicts the spatial map and neural activation time course (blue line) superimposed on corresponding task timings of visual cues (red line) for the dDMN. The dDMN showed the strongest negative correlation (-0.23 on average across all motor task sessions) to visual cue task timings. The second row depicts the spatial maps of the other six networks and their corresponding average correlation with visual cue task timings (median and MAD of correlations in Table S1).

These seven modes were also found in the Brainomics task (spatial maps, mean and standard deviation of neural activation time courses in Figure S22). Similar to MSC tasks, obtained default mode networks negatively correlated with task timings. Figure S22 depicts spatial maps and average neural activation time courses of HMF modes that resembled dDMN, vDMN, PREC, LCEN, RCEN, ASN and VSN in the Brainomics task (among 40 estimated modes). The ASN is known as a mediator between DMNs and other networks such as LCEN, RCEN and VSN. The observed neural activation time courses in our analysis support this hypothesis. The average neural

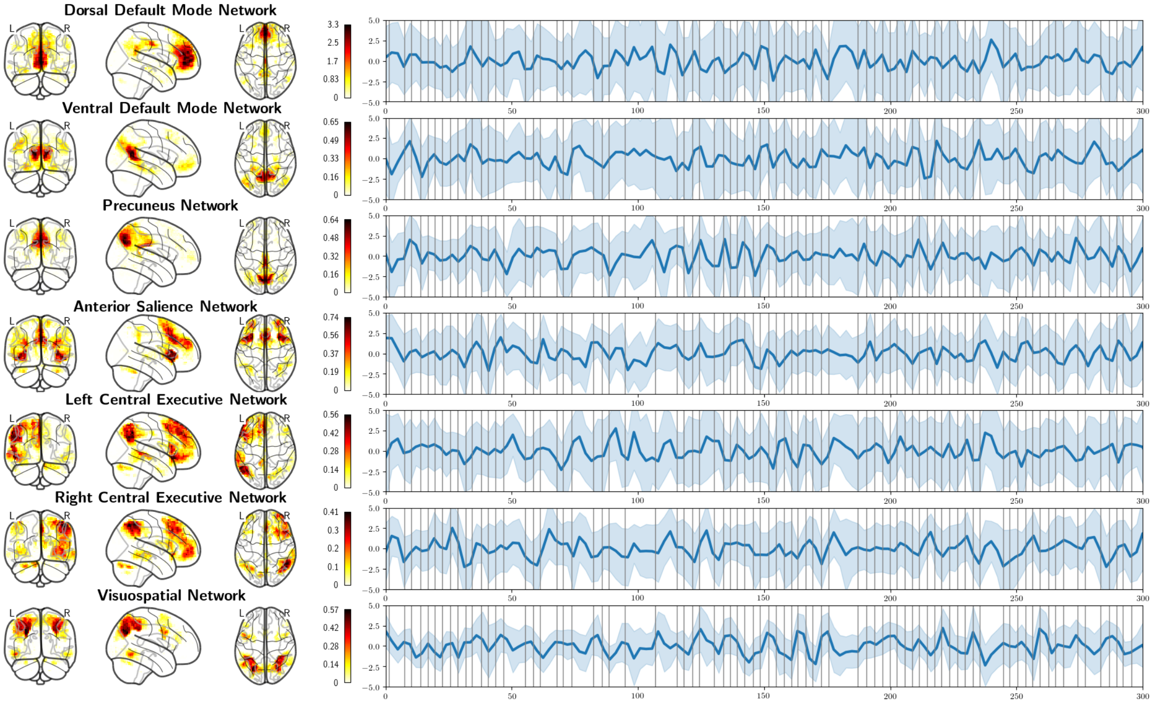

Figure S22: The spatial maps, average and standard deviation for neural activation time courses for modes identified as dDMN, vDMN, PN, LCEN, RCEN, ASN and VSN (among 40 estimated modes).

activation time course (red dashed line) of the ASN across all subjects correlated negatively with the average neural activation time course of dDMN, vDMN and PREC, and correlated positively with the average neural activation time course of LCEN, RCEN, and VSN mode (median and MAD of correlations in Table S3).

Stimuli Type	dDMN	vDMN	PREC	ASN	LCEN	RCEN	VSN
Foot	0.07	-0.03	-0.03	0.05	0.0	-0.02	-0.03
Hand	-0.12	-0.08	-0.06	0.03	0.05	-0.0	0.17
Left Foot	0.03	0.03	-0.01	-0.01	-0.04	-0.04	-0.04
Right Foot	0.03	-0.05	-0.04	0.02	-0.01	0.02	0.02
Left Hand	-0.05	-0.04	-0.06	-0.04	-0.0	0.01	0.09
Right Hand	-0.09	-0.05	-0.04	0.05	0.08	0.03	0.13
Tongue	-0.12	-0.06	0.03	0.19	0.08	0.19	0.14
Block	-0.15	-0.14	-0.1	0.07	0.09	0.1	0.18
Motor Cue	-**0.23**	-0.05	-0.12	0.11	0.04	**0.2**	**0.26**
All Cue	-**0.23**	-0.02	-0.07	0.06	0.08	**0.24**	**0.28**
Rest Cue	0.01	0.08	0.02	-0.02	0.09	0.06	0.03

Table S1: The median correlation between the neural activation time course and corresponding task timings of seven resting-state modes in the MSC motor task (among 40 estimated modes). Values over 0.2 are highlighted in bold.

Stimuli Type	dDMN	vDMN	PREC	ASN	LCEN	RCEN	VSN
Foot	0.12	-0.07	-0.04	0.02	-0.01	-0.09	-0.09
Hand	-0.16	-0.11	-0.14	-0.02	0.07	-0.08	**0.21**
Left Foot	0.03	0.03	-0.02	0.01	-0.05	-0.1	-0.09
Right Foot	0.05	-0.11	-0.05	-0.01	-0.03	0.03	0.01
Left Hand	-0.04	-0.07	-0.12	-0.05	-0.03	-0.03	0.11
Right Hand	-0.1	-0.07	-0.05	0.05	0.07	0.0	0.15
Tongue	-0.18	-0.1	0.09	**0.26**	0.1	**0.26**	0.18
Block	-0.17	-**0.24**	-0.12	0.08	0.14	0.1	**0.2**
Motor Cue	-**0.33**	-0.13	-0.16	0.16	0.02	0.18	**0.33**
All Cue	-**0.29**	-0.02	-0.13	0.14	0.12	**0.24**	**0.33**
Rest Cue	0.07	0.18	0.13	-0.02	0.1	0.13	-0.0

Table S2: The median correlation between BOLD time course and corresponding task design of seven resting-state modes in the MSC motor task (among 40 estimated modes). Values over 0.2 are highlighted in bold.

Stimuli Type	dDMN	vDMN	PREC	LCEN	RCEN	ASN	VSN
Audio	-0.07	-0.07	-0.09	**0.27**	-0.12	-0.01	-0.14
Video	**-0.27**	**-0.27**	-0.08	**0.21**	-0.18	-0.06	0.15
Vertical	0.02	0.02	0.06	-0.14	0.05	-0.03	0.01
Horizontal	0.01	0.07	0.16	-0.07	-0.01	-0.08	-0.02
Left Hand	-0.19	-0.09	-0.09	0.17	-0.08	-0.06	-0.06
Right Hand	-0.1	0.01	0.1	0.13	-0.1	-0.12	-0.08
Phrase	0.06	0.01	**-0.23**	-0.02	0.08	-0.07	-0.19
Calculus	**-0.22**	**-0.32**	0.0	**0.34**	**-0.28**	0.13	**0.27**

Table S3: The median correlation between the neural activation time course and corresponding task timings of seven resting-state modes in the Brainomics data (among 40 estimated modes). Values over 0.2 are highlighted in bold.

Stimuli Type	dDMN	vDMN	PREC	LCEN	RCEN	ASN	VSN
Audio	-0.12	0.01	-0.19	**0.3**	-0.08	0.11	**-0.22**
Video	**-0.3**	**-0.35**	-0.12	0.19	**-0.21**	-0.02	**0.35**
Vertical	0.11	0.09	0.19	**-0.27**	0.08	-0.09	0.07
Horizontal	-0.02	0.04	**0.38**	-0.07	-0.02	-0.17	-0.08
Left Hand	**-0.24**	-0.15	-0.1	0.16	-0.09	-0.02	-0.01
Right Hand	-0.16	0.04	0.1	0.05	-0.06	-0.12	-0.08
Phrase	-0.01	0.06	**-0.3**	0.09	0.11	0.02	-0.17
Calculus	-0.15	**-0.34**	-0.11	**0.32**	**-0.29**	0.19	**0.34**

Table S4: The median correlation between the BOLD time course and corresponding task design of seven resting-state modes in the Brainomics data (among 40 estimated modes).Values over 0.2 are highlighted in bold.

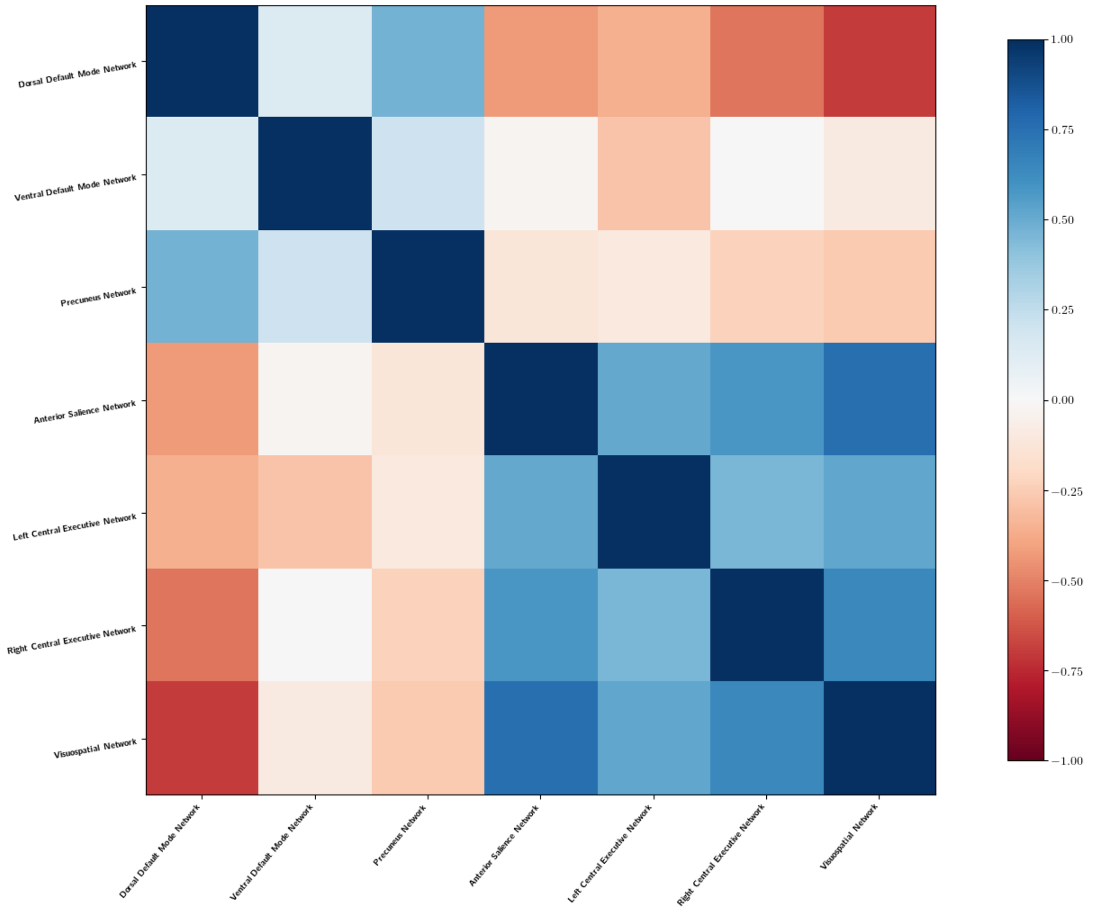

Figure S23: Average of the functional connectivity matrix (average of pairwise correlation between neural activation estimates) between dDMN, vDMN, PN, ASN, LCEN, RCEN and VSN in the MSC motor task runs.

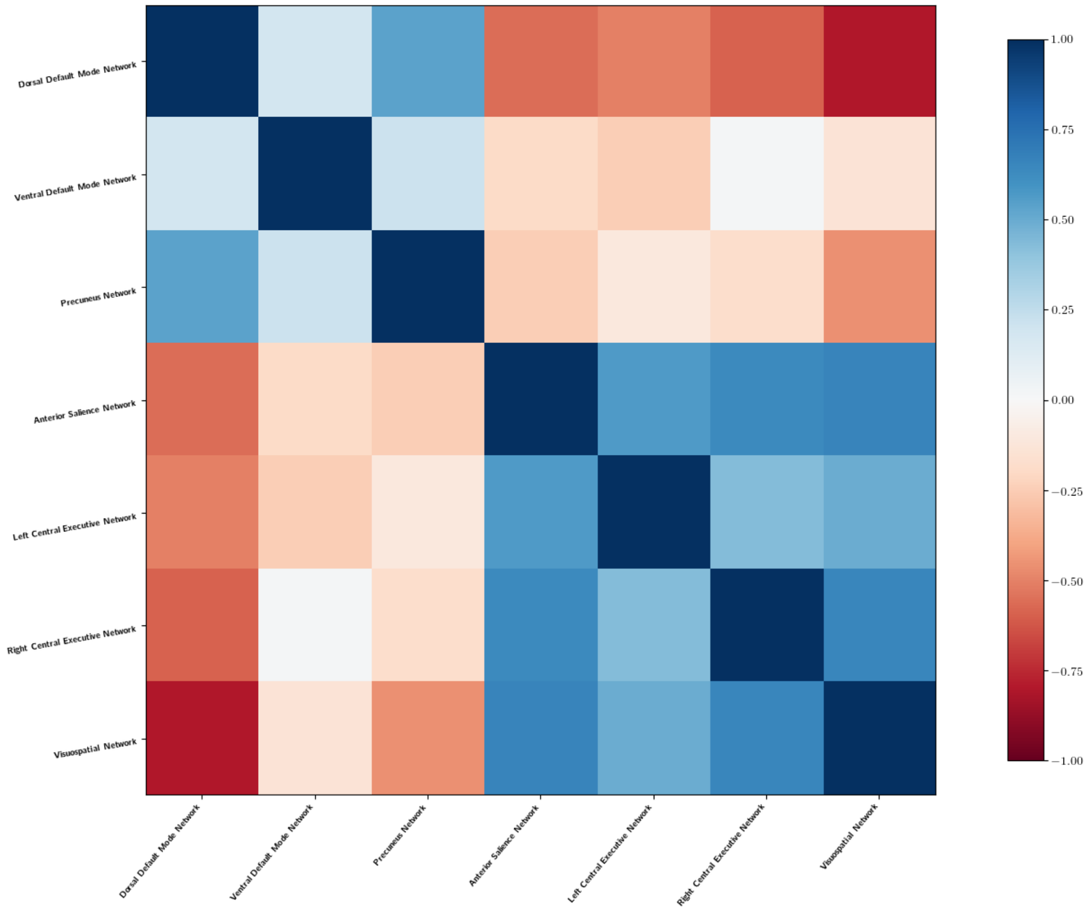

Figure S24: Average of the functional connectivity matrix (average of pairwise correlation between BOLD time course estimates) between dDMN, vDMN, PN, ASN, LCEN, RCEN and VSN in the MSC motor task runs.
